# Perfluoroalkyl and Polyfluoroalkyl Substances Release from Biosolid-Derived Compost

**DOI:** 10.1021/acsomega.5c11002

**Published:** 2026-01-29

**Authors:** Xiangui Huang, Gilboa Arye, Wen Zhang, Avner Ronen

**Affiliations:** † Zuckerberg Institute for Water Research, The Jacob Blaustein Institutes for Desert Research, 108400 Ben-Gurion University of the Negev, Sede-Boqer Campus, Sde-Boqer 8499000, Israel; ‡ French Associates Institute for Agriculture and Biotechnology of Drylands, The Jacob Blaustein Institutes for Desert Research, Ben-Gurion University of the Negev, Sede-Boqer Campus, Be’er Sheva 8499000, Israel; § Department of Civil and Environmental Engineering, 5965 New Jersey Institute of Technology, Newark, New Jersey 07102, United States

## Abstract

This study investigated the release potential and controlling mechanisms of representative per- and polyfluoroalkyl substances (PFAS) of varying carbon-chain lengths from a commercial biosolid-derived compost using sequential leaching with water and saline solutions (10 mM NaCl and 5 mM CaCl_2_). The compost contained >40% organic matter, PFAS concentrations up to 140 ng·g^–1^, and precursor levels below 5 ng·g^–1^. Eluate analyses revealed dissolved organic matter (DOM) concentrations up to 1400 mg·L^–1^ and major ions (Ca, Mg, Na, K, Cl, P, and S) reaching 600 mg·L^–1^, accompanied by PFAS concentrations up to 2600 ng·L^–1^. PFAS desorption followed a biphasic pattern—rapid release within the first hour followed by a slower, sustained phase over 48 h—well described by a first-order two-compartment model with rate constants *k*_1_ = 1–7 h^–1^ and *k*_2_ = 0.001–0.016 h^–1^. Electrostatic interactions dominated the desorption process, as both Na^+^ and Ca^2+^ reduced the fraction of fast-release sites (*F*_1_), with Ca^2+^ showing a stronger suppression due to cation bridging and charge screening. Perfluorohexanoic acid (PFHxA) and perfluorohexanesulfonic acid (PFHxS) were almost completely released within 1 h, whereas perfluorobutanoic acid (PFBA), perfluorobutanesulfonic acid (PFBS), perfluorooctanoic acid (PFOA), and perfluorooctanesulfonate (PFOS) exhibited prolonged desorption associated with interactions with solid organic matter and DOM. DOM-facilitated desorption was evident, as PFAS–DOM complexation enhanced release under certain ionic conditions. Overall, the results reveal the intricate coupling among compost matrix properties, PFAS molecular structure, and eluent chemistry governing PFAS mobility.

## Graphical abstract

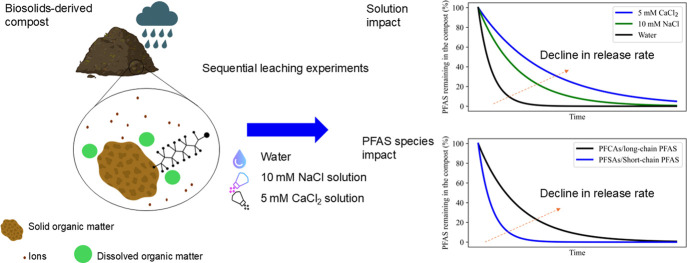

## 1 Introduction

Poly- and perfluoroalkyl substances (PFAS) are synthetic, highly fluorinated aliphatic compounds characterized by a hydrophobic carbon–fluorine tail and a functionalized headgroup,^1^ which typically includes a carboxylic or sulfonic acid group.^2,3^ As a result, PFAS serve as highly effective surfactants and are widely used in various industries, including fabric stain protection, nonstick cookware, and aqueous film-forming foams (AFFF).^3^ PFAS exposure has been linked to adverse human health effects, including immune system suppression, thyroid dysfunction, disruption of insulin regulation, and increased cancer risk.^4^

Due to their widespread use and chemical stability, PFAS are pervasive in environmental matrices such as soil, surface water, and groundwater.^1,5,6^ Specifically, they have been shown to be abundant in landfilled solid waste,^7–9^ discharge of water and biosolids from wastewater treatment plants (WWTPs),^10^ and application of contaminated organic matter (e.g., compost) on agricultural land.^11–15^ Numerous global assessments have demonstrated that WWTPs act as the primary collection and processing hubs for both domestic and industrial effluents, which frequently contain elevated concentrations of PFAS.^16^ Furthermore, PFAS compounds have been detected in municipal wastewater sludge worldwide with concentrations of a few ng·g^–1^ to a few μg·g^–1^.^10,17–19^ PFAS with longer chain lengths showed an enhanced accumulation in the sludge due to their stronger affinity to solid organic matter during the treatment process.^18,20,21^ As a result, biosolids and biosolid-derived compost, commonly applied to farmland as fertilizers, were found to contain elevated PFAS concentrations and a wide range of species diversity. Examples include compost derived from biosolids sources from municipal WWTPs that contained a high PFAS concentration, reaching ΣPFAS of 220 μg·kg^–1^,^12,13,22^ followed by compost made from municipal solid organic waste reaching ΣPFAS of 76 μg·kg^–1^.^11,15^ The lowest levels (ΣPFAS ∼ 0.66 μg·kg^–1^) were found in livestock manure-based compost.^15^

Accordingly, this has raised concerns regarding the potential environmental impacts of compost in agricultural land applications,^11–15^ where PFAS could leach from the compost into the soil following rainfall or irrigation events, posing long-term risks to soil quality, plants, groundwater, and human/animal health.^23,24^ This has been demonstrated through field monitoring,^25^ batch desorption studies,^26^ and column experiment.^27^ For instance, the agricultural application of PFAS-contaminated compost/paper sludge in Germany, mainly in 2000–2008, has resulted in a large-scale PFAS contamination plume (approximately 300 m in length) in the vadose zone.^28,29^ Moreover, PFAS precursors in biosolids can undergo biotransformation, generating terminal PFAS, whereas terminal PFAS themselves are not expected to break down under environmental conditions.^30^ However, data regarding the identity of these precursors, their transformation products, and the underlying reaction pathways remains limited.^1,31^ Accordingly, there are currently no national-level regulatory limits for PFAS in biosolids or compost in either the United States or the European Union, with only a few regional or state authorities (for example, Maine in the United States and selected German states) having implemented concentration-based thresholds for land application.^32,33^

The release of PFAS from biosolids is primarily governed by adsorption and desorption processes, which drive PFAS diffusion to thermodynamic equilibrium between the solid and liquid phases.^34^ The equilibrium is influenced by several factors, including the properties of the compost (e.g., composition, organic matter content, and surface characteristics), the chemistry of the liquid phase (e.g., ionic strength and composition), and the molecular structure of PFAS (e.g., chain length and functional groups).

Specifically, organic matter strongly retains PFAS owing to its high surface area and abundance of reactive functional groups, which enable multiple adsorption mechanisms including hydrophobic interactions, electrostatic attraction, hydrogen bonding, and complexation.^35^ Polyvalent cations further amplify adsorption effects, exerting a stronger influence than monovalent cations.^36,37^ This enhancement is primarily attributed to mechanisms such as cation-induced neutralization of negatively charged sorbent surfaces, screening of anionic PFAS head groups, and reorientation of PFAS molecules at the adsorption interface.^36,37^ Additionally, polyvalent cations can facilitate adsorption through cation-bridging and complexation.^36^ In contrast, the presence of dissolved organic matter (DOM) can promote desorption due to its release from compost and its strong interaction with PFAS molecules.^38^ Although ions and dissolved organic matter are commonly present during PFAS release from compost under field conditions, studies specifically addressing the influence of solution chemistry remain scarce.

In addition, long-chain PFAS are more likely to be adsorbed to the compost due to stronger hydrophobic interactions, whereas short-chain PFAS are readily desorbed. For example, perfluoroalkyl carboxylic acids (PFCAs) with chain lengths of C3–C7 were shown to leach more rapidly than those with longer chain lengths (>C8) under saturated conditions.^39^ Adsorption and desorption processes are also time-dependent and primarily governed by PFAS molecular size, with long-chain PFAS reaching equilibrium faster than short-chain compounds.^40,41^ For example, batch desorption experiments for AFFF-contaminated soil showed that long-chain PFAS reached equilibrium within 48 h, while short-chain PFAS failed to reach equilibrium even after 400 h, highlighting the time-dependent nature of PFAS desorption and the influence of molecular size on their mobility.^40^

Accordingly, this study aims to investigate the release patterns and kinetics of short-chain PFAS such as perfluorobutanoic acid (PFBA), perfluorobutanesulfonate (PFBS), and perfluorohexanoic acid (PFHxA) and long-chain PFAS (perfluorooctanoic acid (PFOA), perfluorohexanesulfonic acid (PFHxS), and perfluorooctanesulfonic acid (PFOS)) from biosolid-derived compost. We investigated how both solution chemistry and PFAS molecular characteristics influence their release behavior from biosolid-derived compost. To quantitatively describe the desorption kinetics, a first-order two-compartment model (FOTWCM) was applied to differentiate between rapid and slow-release domains. This approach enabled evaluation of how ionic strength, cation valency, and PFAS functional groups collectively govern desorption dynamics. The resulting insights are essential for understanding the mechanisms controlling PFAS mobility in compost-amended systems and for assessing their potential to contaminate soil, plants, and groundwater. Moreover, the kinetic pathways and physicochemical interactions underlying PFAS release from biosolid-derived compost under varying ionic conditions were explored, providing a basis for improved environmental risk evaluation and management of compost applications.

## 2 Materials and Methods

### 2.1 Materials

Methanol and acetonitrile for high-performance liquid chromatography-tandem mass spectrometry (HPLC–MS/MS) grade were obtained from Avantor Performance Materials, LLC. Ammonium hydroxide (35%) and glacial acetic acid were purchased from Fisher Scientific (Loughborough, UK). Sodium chloride and calcium chloride were purchased from Sigma. Biosolid-derived compost was supplied by a commercial composting company and was produced from biosolids generated at WWTPs treating municipal wastewater.

Mass-labeled PFAS standard (MPFAC-24ES, lot: PFAC24ES0221), native perfluoroalkyl acids (PFAAs), recovery standard (PFAC-24PAR, lot: PFAC24PAR0321), and mass-labeled injection standard (MPFAC-HIF-IS, lot: MPFACHIFIS0921) were acquired from Wellington Laboratories (UK) for target PFAS analysis. Species’ names and concentrations of MPFAC-24ES, PFAC-24PAR, and MPFAC-HIF-IS are provided in Section S1 of Supporting Information. Superclean ENVI-carb was purchased from Sigma (Germany). CHROMABOND solid phase extraction (SPE) columns (WAX cartridges, REF 730283, sorbent material: polymer-based combination phase) and SPE devices were purchased from MACHEREY-NAGEL (Germany). Milli-Q water (water, pH = 5.0–6.0) was used without further pH adjustment.

### 2.2 Compost Characterization

The evaluated commercial compost was obtained from a composting facility in Israel and is commonly used in agricultural fields and residential gardens. PFAS in the compost was extracted by following the modified US EPA Method 1633 as detailed in Section S2. The compost’s particle size distribution (PSD) was determined by sieving using mesh sizes of 4.0 mm, 2.0 mm, 1.0 mm, and 0.5 mm. Moisture content was assessed by drying the samples in an oven at 105 °C for 12 h, and organic matter content was subsequently quantified by ignition in a muffle furnace at 400 °C for 6 h. The cation exchange capacity (CEC) was measured using the acid washing method.

### 2.3 Sample Preparation and Instrumental Analysis for PFAS

The PFAS extract of the compost and the solution obtained in the release experiment were subsequently concentrated and purified using solid-phase extraction (SPE), as described in Section S3. Following SPE, the collected effluent was evaporated under a gentle nitrogen stream (40 °C, 0.25 L/min) and reconstituted in 1 mL of a water/methanol solution (0.8/0.2, v/v). The reconstituted extract was transferred to a 1.5 mL Eppendorf tube and centrifuged, after which 95 μL of the supernatant was transferred to a 200 μL autosampler vial. Finally, 5 μL of a 200 ng/mL MPFAC-24ES solution was added as an internal standard, and the sample was prepared for instrumental analysis. PFAS analysis was conducted using HPLC (Agilent 1260 Infinity II)-MS/MS (G6465B), as detailed in Section S4.

### 2.4 PFAS Release Experiment and Solution Characterization

To date, there is no standardized US EPA or ASTM leaching protocol designed specifically for PFAS, such as EPA Method 1311 (TCLP), EPA Method 1320 (MEP), and ASTM D 4646, which were originally developed to assess the leachability of metals, inorganic salts, and certain organic contaminants. Given the variability of field conditions, PFAS present in compost may leach slowly under irrigation and light rainfall (0.5–2 mm·h^–1^) or more rapidly under heavy rain (>30 mm·h^–1^).^42^ Assessing the maximum release potential of PFAS from compost is therefore critical. In this study, the sequential leaching approach was employed by referring to EPA Methods 1311 and 1320.^43^ Specifically, our method was adapted from EPA Method 1320 to exhaustively extract releasable PFAS. A solid-to-liquid ratio of 1:10 was selected to ensure exhaustive extraction and sufficient analytical sensitivity. In accordance with U.S. EPA Method 1311, adequate solid material must be used to produce extracts suitable for all required analyses. Preliminary trials showed that a minimum of 8 g of compost was necessary to achieve PFAS concentrations above the HPLC–MS/MS limit of quantification in each leaching step. Therefore, 10 g of compost and 100 mL of leaching solution were used in all experiments.

Because Na^+^, Ca^2+^, and Cl^–^ are the dominant ions present in soil porewater and irrigation sources,^44^ water, 10 mM NaCl, and 5 mM CaCl_2_ solutions (the last two providing 20 mequiv·L^–1^ of ionic strength) were used to assess the influences of ionic composition on PFAS release.^45^ Comparing water with the salt solutions allows evaluation of ionic strength effects, while comparison between NaCl and CaCl_2_ isolates the influence of cation valence. Each elution solution was tested in duplicate (*n* = 2), which provided consistent and reproducible measurements while maintaining feasibility across the elution sequence. The differences in PFAS release among the solutions were evaluated using one-way ANOVA.

Sequential elution experiments were performed using 10 g (dry weight) of compost (particle size ≤ 2.0 mm) and 100 mL of solvents (water, 10 mM NaCl, and 5 mM CaCl_2_) in a 500 mL centrifuge bottle. The bottle was vortexed and subjected to a four-stage leaching sequence consisting of 16 shaking steps: Stage I (10 min × 6), Stage II (1 h × 5), Stage III (6 h × 3), and Stage IV (12 h × 2). After each step, the suspension was centrifuged at 6000*g*, and approximately 80 mL of supernatant was collected. The remaining compost was replenished with fresh solvent to restore the original mass before proceeding to the next step, as described in Section S5.

The eluted solution from each step was analyzed for DOM as indicated by dissolved organic carbon (DOC), total nitrogen (TN), major elements, and PFAS. DOC and TN concentrations were measured using a total organic carbon analyzer (SHIMADZU). DOM composition was further characterized through UV–vis absorbance, and through which SUVA_254_ and *E*_2_/*E*_3_ were calculated to investigate the DOM’s aromaticity and molecular weight distribution. SUVA_254_ is positively correlated with aromaticity, and *E*_2_/*E*_3_ is negatively associated with molecular weight.^46^ Elements, including calcium (Ca), magnesium (Mg), sodium (Na), potassium (K), chloride (Cl), phosphorus (P), and sulfur (S) were quantified by inductively coupled plasma mass spectrometry (ICP–MS) and are used to present the corresponding ions’ concentrations. Additionally, pH and electrical conductivity (EC) were measured using standard pH and EC meters.

Release kinetics were described by a first-order multicompartment model (eqs 1–3),^47^ where the desorption process is divided into different compartments, each governed by distinct rate constants. FOTWCM was employed in this study, which conceptualizes the desorption process by dividing the desorption sites into fast and slow rates. The parameters *F*_1_ and *F*_2_ represent the fraction of PFAS associated with fast and slow-release sites, respectively, while *k*_1_ and *k*_2_ are their corresponding first-order desorption rate constants (h^–1^).

1 $$q_{t}/q_{0}=F_{1}\;\exp\left(-k_{1}t\right)+F_{2}\;\exp\left(-k_{2}t\right)$$

2 $$F_{1}+F_{2}=1,,F_{1}>0,,F_{2}>0$$

3 $$k_{1}>k_{2}>0$$

Temporal PFAS concentration remaining in the compost, *q*_*t*_ (ng·kg^–1^), was calculated using eq 4. This value was derived by subtracting the cumulative mass of PFAS released into the liquid phase from the initial concentration (*q*_0_), which is provided in Table S8.

4 $$q_{t}=q_{0}-\sum c_{t}v_{t}/m$$

where *c*_*t*_ is the PFAS concentration in the liquid phase (ng·L^–1^), *m* denotes the mass of the solid phase (kg), *v*_*t*_ is the volume of the liquid phase (L).

### 2.5 Quality Control

To ensure the quality and integrity of analytical procedures, all PFAS standards were kept at 4 °C in a refrigerator. Compost samples and eluted solutions from the release experiments were stored in a dark room at 4 °C until analysis. PFAS extraction and release experiments were performed in duplicate to assess reproducibility. Two blank samples (Milli-Q water) were included in both the extraction and release experiments to monitor potential contamination. The extraction efficiency (denoted as recovery *R*) for compost was tested in duplicate by spiking compost with 10 μL MPFAC-HIF-IS before extraction. The obtained recovery (Table S4 in Section S2) was used to calculate the real concentration (*C*_ri_) based on the analyzed PFAS concentration (*C*_i_) in the compost through eq 5.

5 $$C_{ri}=C_{i}/R$$

For instrumental analysis, at least three blank and two quality control (QC) samples were included in each batch of analysis to verify instrument cleanliness and analytical precision. Linear calibration curves were generated using least-squares linear regression, with coefficients of determination (*R* ^2^) exceeding 0.95. Most QC samples fell within 100 ± 20% of expected values (Table S4.3), confirming high instrumental accuracy. Additionally, all the instrumental and sample preparation blank samples showed concentrations (Tables S9–S11 in Section 6.3) below the limit of quantification (LOQ = 0.5 ng·mL^–1^), indicating negligible contamination. TOC analyzer and ICP–MS were calibrated monthly. During each analytical batch, a minimum of three blank samples (Milli-Q water) and three to five QC samples were analyzed at the beginning of the run. Additional blanks and QCs were included at the end of the worklist to assess drift and ensure consistency throughout the sequence. High accuracy was obtained for all QC samples (±20%). Blank sample concentrations were below the LOQ, indicating insignificant contamination.

## 3 Results and Discussions

### 3.1 Compost Characteristics

Particle size (diameter) distribution (PSD) analysis of the compost (Figure S4a,b in Section 6.1), revealed three primary fractions: coarse residues (>4 mm), semidecomposed material, including plant residues and biosolid (2–4 mm), and finely degraded organic matter with mineral particles and no plant residues (≤2 mm), comprising 40%, 27%, and 33% of the total mass, respectively.

The water content of the compost fractions ranged from 18% to 25% (Figure S4c). Each fraction exhibited a high proportion of organic matter (Figure S4d), accounting for 49.4 ± 4.0%, 46.1 ± 4.6%, and 36.9 ± 1.4% in the 1–2 mm, 0.5–1 mm, and ≤0.5 mm fractions, respectively. The corresponding cation exchange capacities (CEC) were 26.9 ± 4.9 mequiv 100 g^–1^, 41.8 ± 1.8 mequiv 100 g^–1^, and 39.0 ± 2.0 mequiv 100 g^–1^ (Figure S4e). Both organic matter content and CEC were considerably higher than those typically observed in agricultural soils,^48^ providing abundant reactive sites and nutrient availability. The ≤2 mm fraction was therefore selected for PFAS analysis and release experiments because of its greater homogeneity and representative PSD.

In terms of PFAS composition, PFBS was the most abundant compound in the compost (195.6 ± 6.3 ng g^–1^), followed by PFBA (139.9 ± 3.3 ng g^–1^), PFOS (63.3 ± 6.3 ng g^–1^), PFOA (11.9 ± 0.1 ng g^–1^), PFHxA (7.3 ± 0.2 ng g^–1^), and PFHxS (5.9 ± 0.2 ng g^–1^). These values exceed background concentrations commonly found in uncontaminated soils, which are typically below 5 ng·g^–1^,^49^ but are lower than those found in highly impacted sites, such as surface soil from U.S. military installations contaminated by AFFF, showing up to 8600 ng·g^–1^.^41^ In contrast, surface soil without direct contamination showed a background level of 3.44 ng·g^–1^ for PFOA and 3.13 ng·g^–1^ for PFOS.^50^ In addition to these six primary species, the compost also contained relatively low concentrations of precursors and short-chain species. Specifically, perfluoropentanoic acid (PFPeA) was detected at 0.60 ng·g^–1^, along with 6:2 fluorotelomer sulfonic acid (6:2 FTS, 4.22 ng·g^–1^), 8:2 fluorotelomer sulfonic acid (8:2 FTS, 0.61 ng·g^–1^), and *N*-ethylperfluorooctane sulfonamidoacetic acid (*N*-EtFOSAA, 0.53 ng·g^–1^), indicating a broader PFAS profile and potential for long-term transformation and release.^41^

### 3.2 Release Profiles of DOM and Ions

The release of PFAS from compost occurred in parallel with the release of DOM and major inorganic ions, all of which varied considerably across the three elution conditions. Due to the nature of the compost with high organic matter content, the eluted solutions were found to have high EC and neutral to slightly basic pH (Figure S5 in Section S6.2). The solutes were mainly composed of DOM (up to 1400 mg·L^–1^) and up to 600 mg·L^–1^ ions (Na^+^, K^+^, Ca^2+^, Mg^2+^, and Cl^–^) as shown in Figures S6 and S7, followed by elevated trace levels of PFAS (up to 2600 ng·L^–1^) (Tables S9–S11 in Section S6.3).

#### 3.2.1 Dissolved Organic Matter

DOC exhibited the highest cumulative release among all measured solutes, with final values reaching approximately 475 mg in 10 mM NaCl solution, 400 mg in water, and 340 mg in 5 mM CaCl_2_ solution (Figure S6a). In all eluents, DOC release followed a two-stage pattern: rapid desorption during Stage I, and a slow-release phase in Stages II–IV (1–48 h). The presence of NaCl enhanced DOM solubilization, whereas CaCl_2_ suppressed it throughout the experiment. These differences suggest that Na^+^ increased DOM availability by charge neutralization.^51^ In contrast, Ca^2+^ likely reduced DOM release via cation bridging and DOM aggregation.^52^ Similar trends were seen in leaching experiments from agricultural soil.^53^

DOM quality also evolved over time. Initially, the released DOM was characterized by low molecular weight and hydrophilic fractions, as reflected by low SUVA_254_, high spectral slope ratio (SR), and high *E*_2_/*E*_3_ (Figure S7a–c in Section S6.2).^46^ Over time, SUVA_254_ increased while SR and *E*_2_/*E*_3_ decreased, particularly in the first 6 h, indicating a progressive shift toward more aromatic and higher molecular weight DOM, likely derived from humic and fulvic fractions as indicated by increasing *A*_350_ (Figure S7d).^54^

#### 3.2.2 Inorganic Ions

Cl^–^ exhibited distinct behavior depending on the eluent used (Figure S6b). In water, Cl^–^ was rapidly released during Stage I and then stabilized after the accumulative release mass reached 27 mg. In contrast, when extracted with 10 mM NaCl or 5 mM CaCl_2_, Cl^–^ initially desorbed but was subsequently readsorbed, leading to net negative cumulative values after the first hour. This readsorption under ionic conditions, especially in the presence of Ca^2+^, indicates that the compost contains polar binding sites capable of retaining mobile anions. Such behavior is unusual, given that compost-derived organic matter typically contains a high density of negatively charged functional groups, such as carboxyl and phenolic moieties.^55^

Mg^2+^ showed gradual release in water (∼12 mg) with higher cumulative release in 10 mM NaCl (∼15 mg) and 5 mM CaCl_2_ (∼25 mg) (Figure S6c). This increase reflects cation exchange, in which Na^+^ and Ca^2+^ displaced Mg^2+^ from compost exchange sites. Correspondingly, Ca^2+^, present mainly in the CaCl_2_ treatment, exhibited sustained and substantial adsorption throughout the experiment (Figure S6c). The cumulative uptake reached nearly 190 mg, with no sign of equilibrium. This reflects a strong and persistent affinity of compost for Ca^2+^, as evidenced by the high CEC.

In the NaCl elution, Na^+^ showed continuous adsorption by the compost, with cumulative uptake reaching approximately 30 mg after 48 h. This indicates that Na^+^ from the solution was retained via cation exchange with native K^+^, Mg^2+^, or H^+^ on compost surfaces. The compost’s ability to adsorb Na^+^ may explain the relatively small amount of Na^+^ released in the water eluent. In contrast, in the CaCl_2_ elution, Na^+^ exhibited net release, reaching approximately 30 mg by the end of the experiment, due to displacement by incoming Ca^2+^. K^+^ release followed a similar pattern (Figure S6d). In water, cumulative K^+^ reached ∼15 mg, and increased to ∼47 and ∼42 mg in 10 mM NaCl and 5 mM CaCl_2_.

Since Na^+^ and K^+^ are both monovalent alkali metals with similar ionic radii, they tend to exhibit comparable chemical behavior, making Na^+^ more effective at displacing K^+^. Similarly, Ca^2+^ and Mg^2+^ are both divalent alkaline earth metals with similar charge and size, leading to comparable interactions with exchange sites. As a result, Ca^2+^ was more effective than Na^+^ in replacing Mg^2+^. These patterns reflect the influence of ionic charge and size on competitive ion exchange processes.^56^

Overall, DOM and ions release behavior were strongly shaped by the eluent chemistry. NaCl promoted DOM solubilization and active cation exchange, leading to elevated DOC, Mg^2+^, and K^+^ release and strong Na^+^ uptake. In contrast, CaCl_2_ suppressed DOM release, enhanced the displacement of Mg^2+^, and induced strong retention of both Ca^2+^ and Cl^–^. These trends are attributable to the compost’s high CEC and strong affinity for multivalent ions.

### 3.3 PFAS Release Kinetics

PFAS concentrations in eluted solutions (provided in Tables S9–S11) showed low standard deviations, confirming high experimental reproducibility. Accordingly, mean values were used to calculate the residual PFAS concentrations in the compost. PFAS release profiles were presented as the fraction of remaining PFAS mass in the compost over time. The FOTWCM model was used to fit experimental data, allowing for the distinction between rapid and slow-release phases and the quantification of the release kinetics (Figure 1). Model parameters were optimized by using the Levenberg–Marquardt algorithm (optimized parameters and *R* ^2^ are provided in Table S12 in Section S6.4).

**Figure 1:**
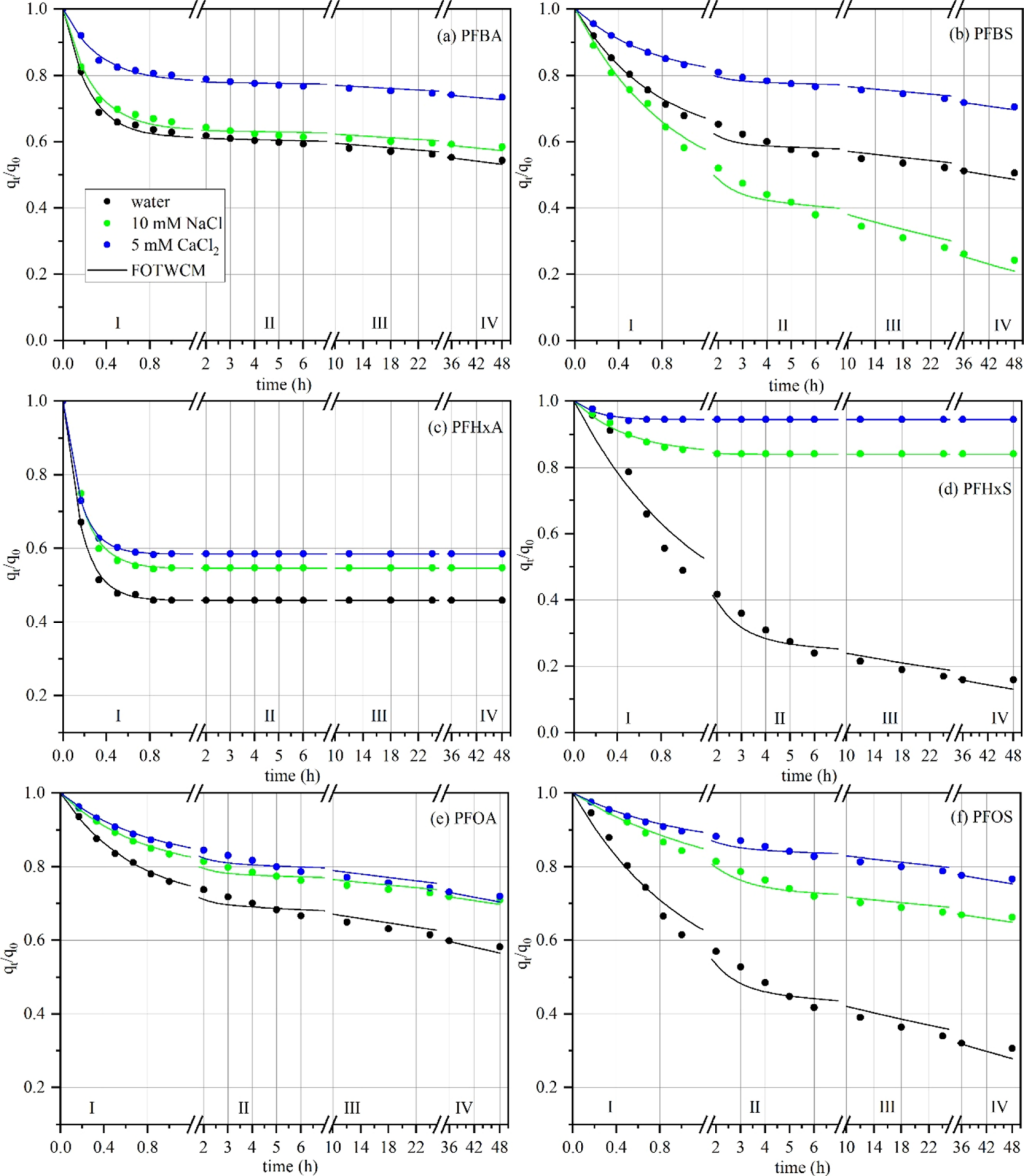
Release kinetics of selected PFAS: (a) PFBA, (b) PFBS, (c) PFHxA, (d) PFHxS, (e) PFOA, and (f) PFOS. The data are presented as the fraction of remaining PFAS (*q*_*t*_/*q*_0_) over time. Each plot includes results from three elution conditions: water, 10 mM NaCl, and 5 mM CaCl_2_.

PFAS release kinetics generally followed a biphasic pattern, with a rapid initial release during the first hour followed by a slower, sustained release over the remaining duration (Figure 1). One-way ANOVA results (Figure S8 in Section S6.5) indicated that, during the fast-release phase, at least two elution conditions differed significantly for most PFAS compounds. In contrast, significant differences were observed in the slow-release phase for most species’ release curves among the three solutions. The specific release profiles for each compound are discussed below.

#### 3.3.1 PFBA and PFBS

Short-chain PFAS compounds, PFBA and PFBS, exhibited similar release patterns characterized by relatively rapid desorption (Figure 1a,b). This was reflected in moderate *F*_1_ values (0.214–0.555) and high fast-phase rate constants (*k*_1_ = 1.152–4.203 h^–1^), followed by high *F*_2_ values (0.445–0.786) and low slow-phase rate constants (*k*_2_ = 0.002–0.016 h^–1^). *F*_1_ values generally declined in the presence of 10 mM NaCl and 5 mM CaCl_2_, except for PFBS in NaCl—indicating reduced availability of fast-desorbing sites under ionic conditions (Figure 2a). In parallel, *F*_2_ increased with the introduction of salts, while *k*_2_ remained relatively stable across all eluents, suggesting that ionic strength and cation valency influence the distribution of PFAS between labile and more strongly retained binding domains, but have limited impact on the slow desorption rate itself.

**Figure 2:**
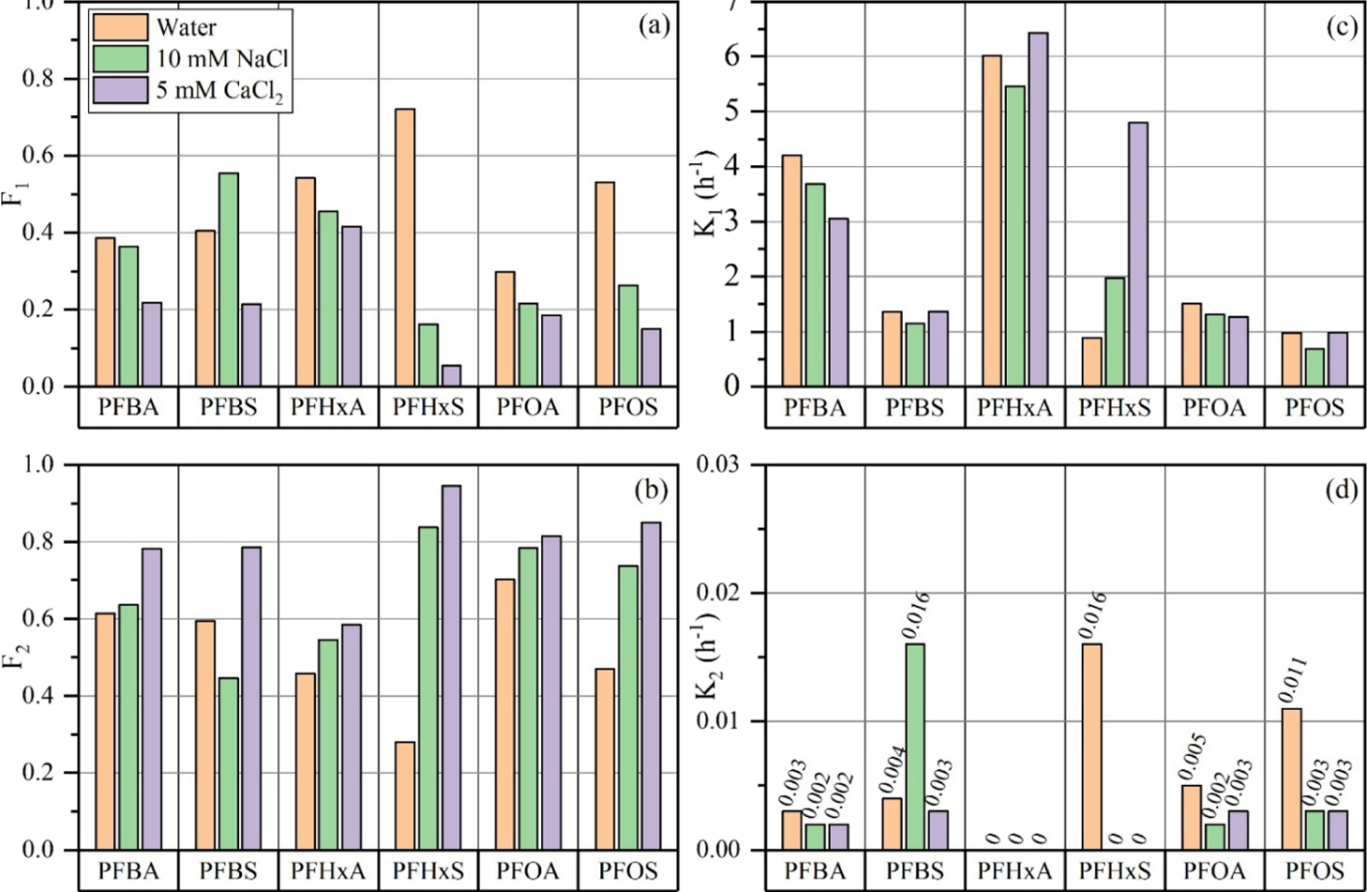
Optimized parameters of the first-order two-compartment model, including: (a) *F*_1_, (b) *F*_2_, (c) *k*_1_, and (d) *k*_2_.

The fast release curves were significantly affected by the presence of 5 mM CaCl_2_, whereas 10 mM NaCl had minimal influence (Figure S8). In contrast, the slow-release curves showed significant differences among all three elution conditions, water, 10 mM NaCl, and 5 mM CaCl_2_. For PFBA, changes in the fast release phase were attributed to variations in both the fast-release fraction (*F*_1_) and the corresponding desorption rate constant (*k*_1_). In the case of PFBS, only *F*_1_ contributed to changes in the fast release behavior. For both compounds, variations in the slow-release fraction (*F*_2_) and slow-phase rate constant (*k*_2_) were responsible for the observed differences in the slow-release curves. PFBS displayed a distinct and unexpected response to ionic strength, deviating from the behavior observed for other PFAS compounds (Figure 1b). In water, PFBS showed an *F*_1_ value of 0.405, which increased to 0.555 in 10 mM NaCl, contrary to the typical trend of reduced desorption with increasing ionic strength. A similar pattern was observed for DOM (Figure S6a in Section S6.2), suggesting that PFBS may be closely associated with DOM, which facilitates its release under monovalent salt conditions. However, in the presence of 5 mM CaCl_2_, PFBS followed the general trend: *F*_1_ declined to 0.214, indicating enhanced retention under divalent cation conditions.

PFBA and PFBS demonstrated faster desorption during the initial hour compared to long-chain PFAS (PFOA and PFOS), although the release rates converged in later stages. These observations highlight the critical role of eluent composition, which can significantly affect the release processes, particularly the presence of divalent cations, in moderating PFAS release, reflected by reductions in *F*_1_ and *k*_1_ and increases in *F*_2_.

#### 3.3.2 PFHxA and PFHxS

Except for PFHxS in water, the release of PFAS compounds (PFHxA and PFHxS) across all eluents was predominantly governed by an initial rapid desorption phase (Figure 1c,d), as evidenced by high *k*_1_ values and negligible or zero *k*_2_ values. PFHxA showed a sharp exponential decline in concentration over time (Figure 1c), with high *F*_1_ values (0.40–0.55) and rapid release rates (*k*_1_ = 5.0–6.5 h^–1^). In contrast, PFHxS displayed a much flatter release curve, with lower *F*_1_ values (0.05–0.15) and slower desorption rates (*k*_1_ = 2.0–5.0 h^–1^). This difference is consistent with the stronger interaction of PFHxS with organic matter, attributed to the higher polarity of the sulfonic acid functional group.^57^ Notably, both PFAS exhibited zero *k*_2_, indicating a lack of measurable release during the slow desorption phase. Additionally, *F*_2_ values suggest that over 40% of the initial PFAS mass remained in the compost, suggesting the presence of irreversible or strongly bound adsorption sites for both PFHxA and PFHxS.

The fast desorption rate constant (*k*_1_) for PFHxA remained consistent across all eluents, ranging from 5.0 to 6.5 h^–1^, and was notably higher than that of PFHxS (0.88–4.80 h^–1^) (Figure 2a). The corresponding *F*_1_ values, which represent the fraction of PFAS associated with fast-desorbing (labile) sites, decreased with increasing cation valency (Figure 2a). For PFHxA, *F*_1_ declined from 0.542 in water to 0.455 in 10 mM NaCl and 0.416 in 5 mM CaCl_2_. PFHxS exhibited a more substantial reduction, with *F*_1_ decreasing from 0.720 in water to 0.162 in NaCl and only 0.055 in CaCl_2_. These results indicate that both compounds are primarily associated with labile adsorption domains, but PFHxS is significantly more sensitive to the presence of divalent cations. The addition of salts, particularly Ca^2+^, appears to shift a portion of PFHxS to more strongly bound or irreversible sites, consistent with prior findings.^58^ The fast release curves of PFHxA were significantly affected by the eluent composition, showing marked differences between water and both 10 mM NaCl and 5 mM CaCl_2_. Statistically significant differences were observed between water and 5 mM CaCl_2_, as well as between 10 mM NaCl and 5 mM CaCl_2_. Additionally, the slow-release curves of PFHxA differed significantly across all three solutions (Figure S8). These changes were primarily driven by a reduction in the fast-release fraction (*F*_1_) and an increase in the slow-release fraction (*F*_2_). In contrast, the variations in PFHxS release behavior, both fast and slow phases, were attributed to simultaneous changes in both the fractional distributions (*F*_1_ and *F*_2_) and their respective desorption rate constants (*k*_1_ and *k*_2_).

#### 3.3.3 PFOA and PFOS

The release of long-chain PFAS compounds, PFOS and PFOA, into water followed a two-phase desorption pattern: an initial rapid release phase followed by a slower, sustained release (Figure 1e,f). This behavior was characterized by a relatively low fast-release fraction (*F*_1_ = 0.150–0.531) and a high rate constant for the fast phase (*k*_1_ = 0.688–1.506 h^–1^), followed by a larger slow-release fraction (*F*_2_ = 0.469–0.850) and a low rate constant for the slow phase (*k*_2_ = 0.002–0.011 h^–1^). When the eluent was changed to 10 mM NaCl and 5 mM CaCl_2_, the *F*_1_ was decreased, while the release rate constants remained largely unchanged. This suggests a reduction in the rapid desorption phase (Stage I), likely due to the reduced mobilization of PFAS that are weakly bound to the external surfaces of compost particles. According to the ANOVA test, the fast-release fraction (*F*_1_) of PFOA significantly decreased when the eluent was changed from water to 5 mM CaCl_2_, leading to substantial alterations in the corresponding fast release curves. In the case of PFOS, *F*_1_ significantly declined not only from water to 10 mM NaCl but also to 5 mM CaCl_2_, resulting in further pronounced changes in its fast release behavior. These shifts were accompanied by an increase in the slow-release fraction (*F*_2_) and a decrease in the corresponding desorption rate constant (*k*_2_), yielding significantly different slow-release curves for PFOA between water and both salt solutions, and for PFOS across all three elution conditions (Figure S8).

### 3.4 Cumulative Released Mass

The cumulative mass proportions of PFAS released at the end of the experiment are shown in Figure 3. The solution composition, particularly the presence of divalent cations, influenced cumulative PFAS release (Figure 3a), which is aligned with the results of the ANOVA test (Figure S8). In water, the overall release was high, especially for perfluorosulfonic acids (PFSAs), including PFBS (49.39%), PFHxS (84.07%), and PFOS (69.35%). Perfluoroalkyl carboxylic acids (PFCAs), such as PFHxA (54.06%), PFBA, and PFOA (both ∼45%), exhibited slightly lower cumulative releases.

**Figure 3:**
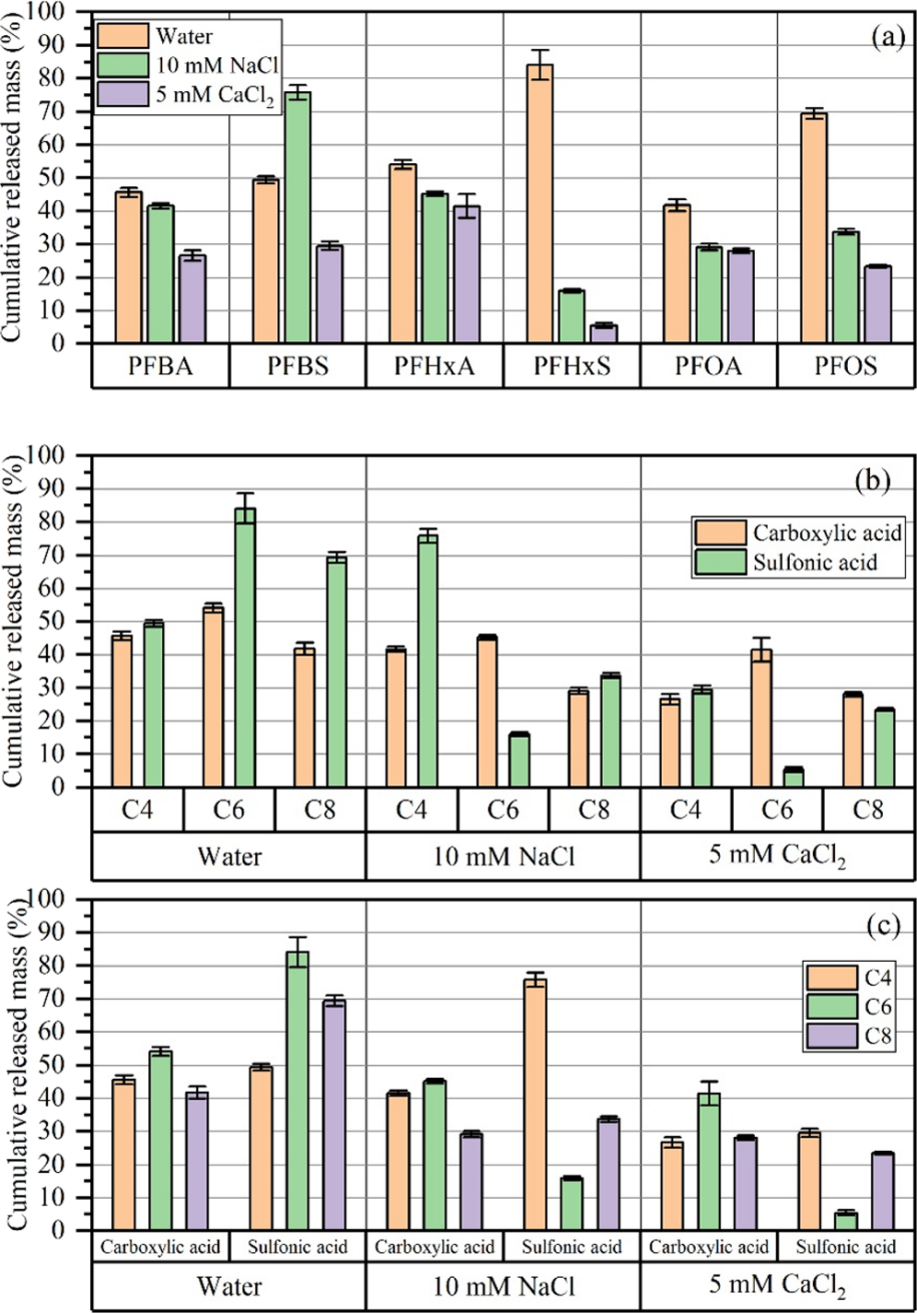
Cumulative PFAS mass released from compost under different elution conditions, organized by (a) the effect of solution cations on individual PFAS compounds; (b) the influence of PFAS functional groups (carboxylic acid vs sulfonic acid) across different chain lengths and eluents; (c) the effects of chain length on PFAS release in each solution.

In the presence of monovalent cations (10 mM NaCl), most PFAS exhibited suppressed desorption, with the cumulative release dropping to 40–45% for PFBA and PFHxA, 29.5% for PFOA, and 33.7% for PFOS. This may result from the compression of the electrostatic double layer and the masking of surface charge, which can lead to limited rejection between the negatively charged PFAS and organic matter.^59^ PFBS exhibited unique behavior as described in Section 3.3.3, resulting in a higher cumulative release in the presence of monovalent ions, reaching 76.1% (Figure 3a).

In solutions containing divalent cations (5 mM CaCl_2_), PFAS desorption was universally suppressed, with all compounds showing the lowest cumulative release among the three eluents. Final values fell to 26.94% for PFBA, 41.42% for PFHxA, 27.59% for PFOA, 29.57% for PFBS, 23.49% for PFOS, and 5.71% for PFHxS. The significant suppression, especially for PFHxS, indicates a strong interaction between the compost matrix and PFAS under Ca^2+^-rich conditions.^60^

The impacts of functional group revealed that PFSAs (PFBS, PFHxS, and PFOS) were more sensitive to eluent composition than PFCAs (PFBA, PFHxA, and PFOA) (Figure 3b). In water, PFSAs exhibited higher cumulative release percentages than PFCAs of the same chain length, likely due to their stronger interaction with DOM, as the sulfonate functional group is more polar.^57^ However, this trend was disrupted upon the addition of NaCl and CaCl_2_, leading to the cumulative release percentages of PFHxA and PFOA being larger than PFHxS and PFOS in 10 mM CaCl_2_.

When comparing the cumulative released mass by PFAS chain length, short-chain compounds such as PFBA and PFBS desorbed more rapidly and reached higher cumulative release levels than the long-chain compound PFOA in water, consistent with their lower hydrophobicity (Figure 3c).^61^ However, PFBS exhibited lower cumulative release than PFHxS and PFOS, despite its shorter chain length, which deviates from the expected trend and suggests that additional factors, such as functional group interactions or DOM association, may influence its desorption behavior.

In the monovalent solution (10 mM NaCl), most results were consistent with the hydrophobicity order: short-chain PFCAs (PFBA and PFHxA) released more than long-chain (PFOA), and for PFSAs, only PFBS > PFOS. It is possibly due to the ability of Na^+^ to screen the electrostatic forces between PFAS and anionic sorbents, enhancing the hydrophobic interaction between long-chain PFAS and the solid compost particles.^59^ The difference between long and short-chain PFAS significantly diminished in the presence of divalent cations (5 mM NaCl_2_), as Ca^2+^ can promote the adsorption of PFCAs and PFSAs through cation bridging, leading to limited desorption.^60^

### 3.5 Mechanisms Controlling PFAS Desorption

The proposed mechanisms discussed in this section are based on observed desorption patterns and are intended as informed assumptions rather than definitive conclusions. They aim to interpret the experimental results by considering PFAS physicochemical properties, compost composition, and the influence of ionic strength and cation valency on PFAS mobility. Further molecular-level investigations would be required to confirm these interpretations. To support the evaluation of potential desorption mechanisms, Pearson correlation coefficients (PCCs) were calculated between PFAS release profiles and the release of other solutes (Figure S9 in Section S6.6). These correlations help identify possible comobilization pathways or shared binding domains.

Accordingly, the presence of cations, both monovalent and divalent, played a key role in suppressing PFAS desorption. This suppression appears to operate through two main mechanisms: (1) promoting PFAS retention on compost organic matter by cation bridging and surface charge neutralization, and (2) limiting the release of DOM, which could otherwise enhance PFAS mobility. Divalent cations (Ca^2+^) exerted a stronger effect than monovalent (Na^+^), likely due to their capacity to form ionic bridges between the negatively charged PFAS head groups and the compost matrix.^36^ These observations suggest that electrostatic interactions are the primary mechanism governing PFAS retention and desorption in compost.

High positive PCCs were observed between PFAS and mobile solutes such as DOM, Na^+^, Cl^–^, and K^+^, indicating comobilization likely driven by surface-associated processes (Figure 4). In contrast, near-zero or negative PCCs with Ca^2+^ and Mg^2+^ suggest that these less soluble divalent cations are released more gradually and are not directly involved in the early desorption of PFAS. These patterns underscore the influence of solubility and ionic competition during the initial release phase, illustrating that PFAS desorption from compost is governed by more dynamic and transient interactions than those observed in conventional batch adsorption/desorption studies using permanent sorbents.

**Figure 4:**
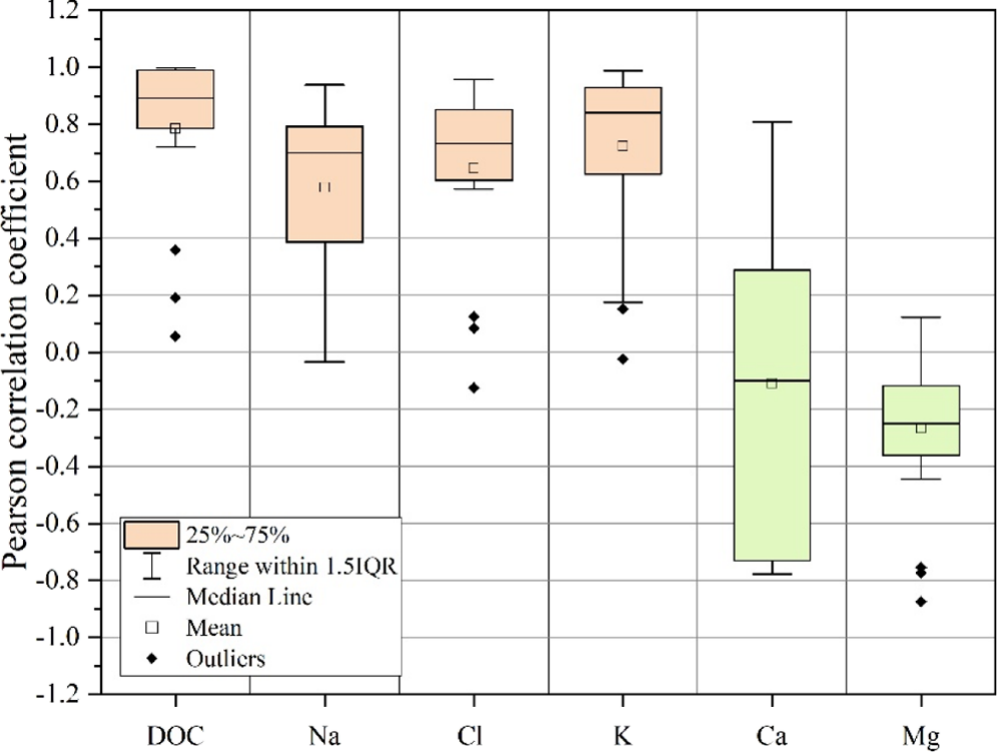
Pearson correlation coefficients illustrating the relationships between PFAS release kinetics, DOM, and major ions.

DOM emerged as a key factor influencing PFAS mobility, exhibiting the strongest correlation with PFAS release among all measured solutes (Figure 4). This strong association indicates that PFAS desorption is closely tied to DOM leaching throughout the experiment. Considering the compost’s origin in wastewater treatment sludge, where PFAS and DOM are coprecipitated, PFAS molecules are likely not only adsorbed onto the organic matter surfaces but also embedded within the organic matrix.^36^ Consequently, the rapid release observed in Stage I can be attributed to the dissolution of labile organic matter at the surface of compost particles, while the prolonged slow-release phase is governed by the gradual breakdown of more recalcitrant organic fractions. This transition from fast dissolution to slower diffusion reflects a shift in the dominant desorption mechanism, with cation effects becoming more pronounced during the later stages, as proved by the one-way ANOVA test.

The influence of hydrophobic interactions on PFAS desorption was inferred from the cumulative release patterns of short-chain (PFBA and PFBS) and long-chain (PFOA and PFOS) compounds (Figure 3c). Under water and monovalent salt conditions, long-chain PFAS exhibited lower release, consistent with stronger hydrophobic retention. However, these differences diminished in the presence of divalent cations (Ca^2+^), indicating that electrostatic interactions and cation bridging became the dominant forces, overshadowing hydrophobic effects. PFAS functional groups also played a significant role. In water, PFSAs (PFBS, PFHxS, and PFOS) were more readily desorbed than PFCAs (PFBA, PFHxA, and PFOA) (Figure 3b), likely due to the higher polarity and stronger hydrogen-bonding capacity of the sulfonic acid group.^57^ However, the presence of salts, particularly divalent cations, reduced these functional group-dependent differences, further emphasizing the overriding influence of ionic interactions in PFAS desorption.

Notably, PFBS exhibited an unexpected response in 10 mM NaCl, where its release was enhanced relative to water (Figure 1a). This deviation is likely due to PFBS’s strong affinity for DOM. Na^+^ is known to promote DOM solubilization, which in turn facilitates PFBS release through cotransport mechanisms.

The compost used in this study, with an organic matter content exceeding 40%, generated DOM concentrations above 2500 mg·L^–1^, creating conditions under which PFAS hydrophobicity and mobility can be substantially altered by solubilization within the DOM matrix. This interpretation is supported by surface tension measurements, which revealed that the surface tension of a 10 mg·L^–1^ PFOS solution increased from 50.93 mN·m^–1^ to 65.42 mN·m^–1^ upon addition of 10 mg·L^–1^ DOM (extracted from the same compost), indicating strong PFOS–DOM interactions that reduce surface activity and promote solubilization.

## 4 Environmental Implications and Conclusions

This study assessed the maximum PFAS release potential from compost based on sequential leaching experiment. The results demonstrate that biosolid-derived compost can act as a persistent and complex source of PFAS to agricultural systems. The release behavior of six representative PFAS compounds showed clear distinctions between short-chain and long-chain molecules under different elution conditions. Sequential leaching experiments revealed biphasic desorption kinetics across all eluents, with PFHxA and PFHxS exhibited rapid desorption during the first hour, while PFBA, PFBS, PFOA, and PFOS displayed both fast and slow-release phases over a 48 h period. Eluent composition played a critical role, as the presence of salts, particularly divalent cations like Ca^2+^, substantially suppressed PFAS release. This suppression is attributed to mechanisms such as cation bridging, electrostatic screening, and reduced DOM solubilization.^52,59^ Furthermore, PFAS physicochemical properties such as chain length and functional group polarity were found to influence release dynamics, with PFSAs showing greater sensitivity to eluent composition than PFCAs.

The FOTWCM provides valuable insight into environmental behavior. Extrapolation of model parameters suggests that PFOA and PFOS may continue to leach from compost over hundreds to more than a thousand hours, depending on the ionic composition of the infiltrating water. As these are observed under a high solid-to-liquid ratio, this raises concern for long-term (e.g., several years) contamination risks in most field conditions, particularly in regions that rely on saline or reclaimed water for irrigation. This is especially relevant in arid and semiarid areas, where suppressed desorption can delay PFAS release but prolong exposure.^62^ Moreover, compost is not a chemically or biologically inert matrix. Once applied to soils, microbial degradation, root exudates, and abiotic processes such as UV exposure, redox cycling, and physical fragmentation alter the compost structure, potentially increasing the availability of previously sequestered PFAS. These processes not only enhance the release of strongly sorbed terminal PFAS but also promote the transformation of precursors, which further contributes to long-term contamination.^63^

In conclusion, this study highlights the necessity for environmental and regulatory assessments to move beyond static concentration metrics and consider dynamic desorption processes, DOM interactions, and precursor transformation pathways. The persistence of both freely available and strongly bound PFAS fractions, combined with compost degradation and ionic interactions in soil, underscores the importance of monitoring and managing biosolid-derived compost applications. Regulatory frameworks should incorporate long-term kinetic data and transformation potential when evaluating PFAS mobility and exposure risks in agricultural systems. In future work, PFAS release under acidified conditions representative of rainwater will be examined, and long-term or interval-based leaching experiments will be conducted to evaluate precursor transformation.

## Supplementary Material



## Notes

The Supporting Information includes detailed descriptions of the PFAS standards used for quantitative analysis, as well as the protocols for sample preparation and instrumental analysis, including instrument configurations and key operational parameters. It also provides the experimental procedures for the PFAS release experiments conducted in this study. These materials are intended to enable full reproducibility of the experiments and analyses by other researchers. Finally, all data sets generated in this study are provided in the final section in the form of schematic illustrations and raw data tables, which support and complement the results and discussion presented in the main manuscript (PDF)
