# Supplementary material for: Perfluoroalkyl and Polyfluoroalkyl Substances Release from Biosolid-Derived Compost
Source: ACS Omega. 2026 Jan 29;11(6):10062–72. doi: 10.1021/acsomega.5c11002 (PMC12917841; doi:10.1021/acsomega.5c11002)
Supplement: Supplementary file 1 [file ao5c11002_si_001.pdf]

Supporting Information

Perfluoroalkyl and Polyfluoroalkyl Substances Release from Biosolid-Derived Compost

Xiangui Huang[a], Gilboa Arye[b], Wen Zhang[c], Avner Ronen[a]*

[a] Zuckerberg Institute for Water Research, The Jacob Blaustein Institutes for Desert Research, Ben-Gurion University of the Negev, Sede-Boqer Campus 8499000, Israel

[b] French Associates Institute for Agriculture and Biotechnology of Drylands, The Jacob Blaustein Institutes for Desert Research, Ben-Gurion University of the Negev, Sede-Boqer Campus 8499000, Israel

[c] Department of Civil and Environmental Engineering, New Jersey Institute of Technology, Newark, New Jersey 07102, the United States

* Corresponding author: Avner Ronen avnerr@bgu.ac.il

S1. PFAS Standards

The mass-labeled PFAS standard (MPFAC-24EC) consists 10 mass-labeled (^{13}C) perfluoroalkyl carboxylic acids (C_4-C_{12} and C_{14}), three mass-labeled (^{13}C) perfluoroalkyl sulfonates (C_4, C_6, C_8), three mass-labeled (^{13}C) telomer sulfonates (4:2, 6:2, 8:2), and two mass-labeled (^2H) perfluorooctane sulfonamido acetic acids, and perfluoro-1-($^{13}C_8$) octanesulfonamide. Their full name, acronyms, and concentrations are introduced in **Table 1**.

Table S1. The name, acronym, and concentration in mass-labeled PFAS standard (MPFAC-24EC)

	Compound	Acronym	Concentration (ng/mL)
	**Perfluoroalkyl carboxylic acids**		
1	Perfluoro-n-($^{13}C_4$) butanoic acid	MPFBA	1000
2	Perfluoro-n-($^{13}C_5$) pentanoic acid	M5PFPeA	1000
3	Perfluoro-n-(1, 2, 3, 4-$^{13}C_5$) hexanoic acid	M5PFHxA	1000
4	Perfluoro-n-(1, 2, 3, 4-$^{13}C_4$) heptanoic acid	M4PFHpA	1000
5	Perfluoro-n-($^{13}C_8$) octanoic acid	M8PFOA	1000
6	Perfluoro-n-($^{13}C_9$) nonanoic acid	M9PFNA	1000
7	Perfluoro-n-(1, 2, 3, 4, 5, 6-$^{13}C_6$) decanoic acid	M6PFDA	1000
8	Perfluoro-n-(1, 2, 3, 4, 5, 6, 7-$^{13}C_7$) undecanoic acid	M7PFUdA	1000
9	Perfluoro-n-(1, 2-$^{13}C_2$) dodecanoic acid	MPFDoA	1000
10	Perfluoro-n-(1, 2-$^{13}C_2$) tetradecanoic acid	M2PFTeDA	1000
11	Perfluoro-1-($^{13}C_8$) octanesulfonamide	M8FOSAA	1000
	**Perfluorooctane sulfonamido acetic acids**		

12	N-methyl-d3-perfluoro-1-octanesulfonamidoacetic acid	D3-N-MePOSAA	1000
13	N-ethyl-d5-perfluoro-1-octanesulfonamidoaxetic acid	D5-N-EtFOSAA	1000
	**Perfluoroalkyl sulfonates**		
14	Sodium perfluoro-1-(2,3,4-$^{13}C_3$) butanesulfonate	M3PFBS	1000
15	Sodium perfluoro-1-(1,2,3-$^{13}C_3$) hexanesulfonate	M3PFHxS	1000
16	Sodium perfluoro-1-($^{13}C_8$) octanesulfonate	M8PFOS	1000
	**Telomer sulfonates**		
17	Sodium 1H, 1H, 2H, 2H-perfluoro-1-(1,2-$^{13}C_2$) hexanesulfonate	M2-4:2FTS	1000
18	Sodium 1H, 1H, 2H, 2H-perfluoro-1-(1,2-$^{13}C_2$) octanesulfonate	M2-6:2FTS	1000
19	Sodium 1H, 1H, 2H, 2H-perfluoro-1-(1,2-$^{13}C_2$) decanesulfonate	M2-8:2FTS	1000

Notes: concentrations have been rounded to three significant figures.

The native PFAS standard (PFAC-24PRA) includes 11 native linear perfluoroalkyl carboxylic acids (C4-C14), seven native perfluoroalkyl sulfonates, three native telomer sulfonates, two native perfluoro octanesulfonamido acetic acids, and perfluoro-1-octanesulfonamide. Their name, acronyms, and concentrations are given in **Table S2**.

Table S2. The name, acronym, and concentration in the native PFAS standard (PFAC-24PRA)

	**Compound**	**Acronym**	**Concentration (ng/mL)**
	**Perfluoroalkyl carboxylic acids**		
1	Perfluoro-n-butanoic acid	PFBA	2000
2	Perfluoro-n-pentanoic acid	PFPeA	2000

3	Perfluoro-n-hexanoic acid	PFHxA	2000
4	Perfluoro-n-heptanoic acid	PFHpA	2000
5	Perfluoro-n-octanoic acid	PFOA	2000
6	Perfluoro-n-nonanoic acid	PFNA	2000
7	Perfluoro-n-decanoic acid	PFDA	2000
8	Perfluoro-n-undecanoic acid	PFUdA	2000
9	Perfluoro-n-dodecanoic acid	PFDoA	2000
10	Perfluoro-n-tridecanoic acid	PFTrDA	2000
11	Perfluoro-n-tetradecanoic acid	PFTeDA	2000
12	Perfluoro-1-octanesulfonamide	FOSA	2000
	**Perfluoro octanesulfonamido acetic acids**		
13	N-methylperfluoro-1-octanesulfonamidoacetic acid	N-MeFOSAA	2000
14	N-ethylperfluoro-1-octanesulfonamidoaxetic acid	N-EtFOSAA	2000
	**Perfluoroalkyl sulfonates**		
15	Sodium perfluoro-1-butanesulfonate	L-PFBS	2000
16	Sodium perfluoro-1-pentanesulfonate	L-PFPeS	2000
		PFHxSK (linear)	1620
17	Potassium perfluorohexanesulfonate	PFHxSK (branched isomers)	378
18	Sodium perfluoro-1-heptanesulfonate	L-PFHpS	2000
		PFOSK (linear)	1580
19	Potassium perfluorooctanesulfonate	PFOSK (branched isomers)	422
20	Sodium perfluoro-1-nonanesulfonate	L-PFNS	2000
21	Sodium perfluoro-1-decanesulfonate	L-PFDS	2000
	**telomer sulfonates**		

22	Sodium 1H, 1H, 2H, 2H-perfluoro-1-hexanesulfonate	4:2FTS	2000
23	Sodium 1H, 1H, 2H, 2H-perfluoro-1-octanesulfonate	6:2FTS	2000
24	Sodium 1H, 1H, 2H, 2H-perfluoro-1-decanesulfonate	8:2FTS	2000

Notes: concentrations have been rounded to three significant figures. For perfluoroalkylsulfonates, the given concentration represents the content of their salt form.

The mass-labeled PFAS injection standard (MPFAC-HIF-IS) consists of five mass-labeled (^{13}C) perfluoroalkycarboxylic acids (C_4, C_6 and C_8 - C_{10}), two mass-labeled (^{13}C) perfluoroalkysulfonates (C_6 and C_8). Their name, acronyms, and concentrations are given in Table S3.

Table S3. The name, acronym, and concentration in mass-labeled injection PFAS standard (MPFAC-HIF-IS)

	**Compound**	**Acronym**	**Concentration (ng/mL)**
	**Perfluoroalkyl carboxylic acids**		
1	Perfluoro-n-(2, 3, 4-$^{13}C_3$) butanoic acid	M3PFBA	1000
2	Perfluoro-n-(1, 2-$^{13}C_2$) hexanoic acid	MPFHxA	500
3	Perfluoro-n-(1, 2, 3, 4-^{13}C4) octanoic acid	MPFOA	500
4	Perfluoro-n-(1, 2, 3, 4,5-$^{13}C_5$) nonanoic acid	MPFNA	250
5	Perfluoro-n-(1, 2-$^{13}C_2$) decanoic acid	MPFDA	250
	**Perfluoroalkyl sulfonates**		
6	Sodium perfluoro-1-hexane($^{18}O_2$) sulfonate	MPFHxS	500

7	Sodium perfluoro-1-(1, 2, 3, 4-$^{13}C_4$) octanesulfonate	MPFOS	500

S2. PFAS Extraction Protocol for Compost

The extraction procedure follows US EPA Method 1633 and consists of the following steps:

(1) Homogenized dry compost (2 g) is placed in a 50 mL polypropylene Falcon tube, spiked with 20 µL of mass-labeled injection standard, and allowed to stand for 24 hours prior to extraction. (2) A volume of 10 mL of 0.3% methanolic ammonium hydroxide is added. The mixture is vortexed and sonicated at 30 °C for one hour, then mixed by end-over-end rotation for two hours. (3) The sample is centrifuged at 7000 rpm for 15 minutes, and the supernatant is carefully transferred into a clean 50 mL polypropylene tube, avoiding any solid residue. (4) The remaining solid is retained, and steps (2) and (3) are repeated three times. (5) All resulting supernatants are combined in a single 50 mL Falcon tube to form the compost extract. (6) To the combined extract, 50 mg of ENVI-Carb is added. The tube is vortexed briefly, centrifuged at 8000 rpm for 15 minutes, and the clarified liquid is transferred to a 200 mL polypropylene bottle. (7) Milli-Q water is added to bring the aqueous content to approximately 80%. The pH is adjusted to 7.0 using 2 M HCl. (8) The final extract is then concentrated and purified using solid phase extraction (SPE). The recovery of this protocol was presented in Table S2.1.

Table S4. Recovery of the PFAS extraction protocol

(n=2)	**PFBA**	**PFBS**	**PFHxA**	**PFHxS**	**PFOA**	**PFOS**
**Recovery (mean, %)**	64.91	59.00	61.95	54.93	50.26	43.08
**S.D.**	7.37	3.44	7.10	5.77	2.90	0.20
**RSD (%)**	11.36	5.83	11.46	10.50	5.78	0.46

Notes: RSD =100×(S.D./mean), **S.D.** is standard deviation.

S3. Solid Phase Extraction (SPE)

S3.1. Instrument

The solid phase extraction (SPE) is carried out using a 12-position vacuum manifold connected to an air pump with adjustable pressure control (Figure S1). The aqueous solution is loaded into the SPE cartridges and drawn through by vacuum, with the eluent collected in a plastic container placed inside the manifold.

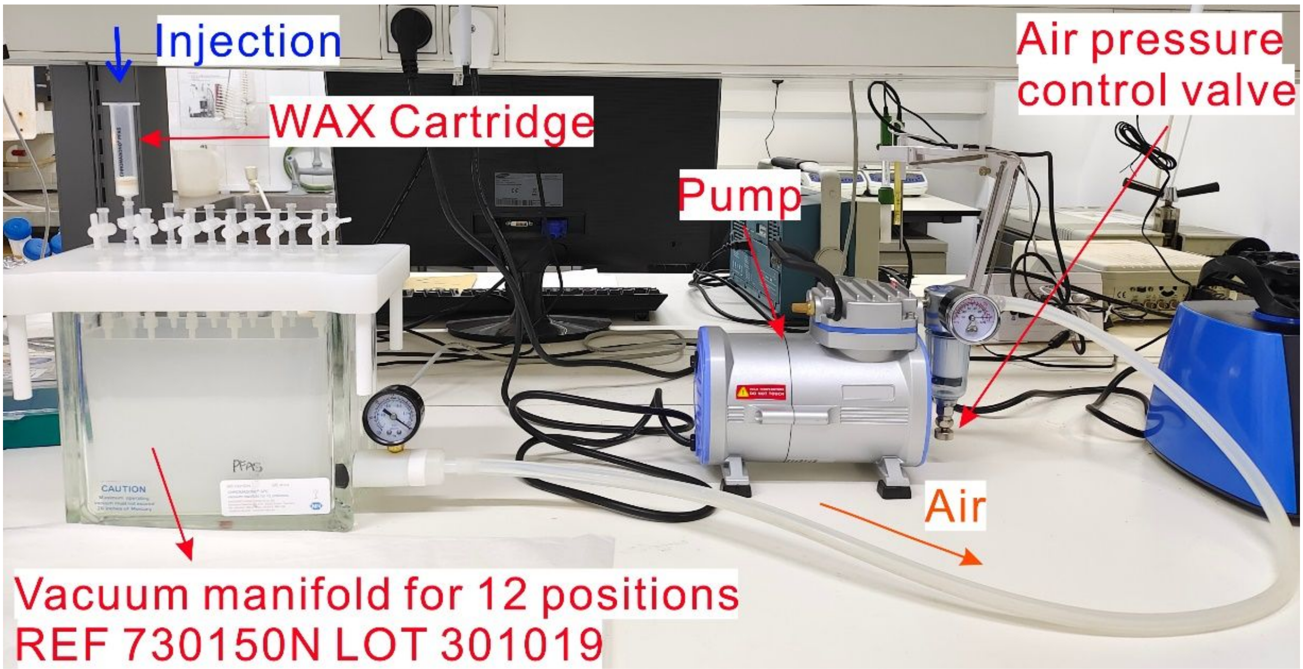

Figure S1. Instruments for performing SPE

S3.2. Chemicals

The following chemicals were used in the solid phase extraction process:

(1) 0.1% methanolic ammonium hydroxide solution (prepared by dissolving NH_4OH in methanol);

(2) 0.1 M acetic acid buffer, adjusted to pH ~4; (3) Milli-Q water; (4) Methanol.

S3.3. Method

(1) Weak anion exchange (WAX) cartridges were pre-conditioned sequentially with 10 mL of 0.1% methanolic ammonium hydroxide, followed by 10 mL of methanol, and then 10 mL of Milli-Q water.

(2) The pre-treated compost extract was loaded onto the WAX cartridge at a flow rate of 3–5 mL/min. The sample container was rinsed with 5 mL of Milli-Q water, and the rinse was passed through the same cartridge.

(3) Once the entire sample had passed through the cartridge, it was washed with 5 mL of 0.1 M acetic acid buffer at a flow rate of 6 mL/min.

(4) The cartridge was dried under vacuum at -0.1 bar for 30 seconds.

(5) All flow-through fractions from steps (1) through (4) were discarded.

(6) Target analytes (PFAS) were eluted from the cartridge using 6 mL of 0.1% methanolic ammonium hydroxide at a flow rate of 3 mL/min. The eluate was collected in a 15 mL polypropylene tube.

S4. HPLC-MS/MS

The employed HPLC-MS/MS system is schematically represented in Figure S2.

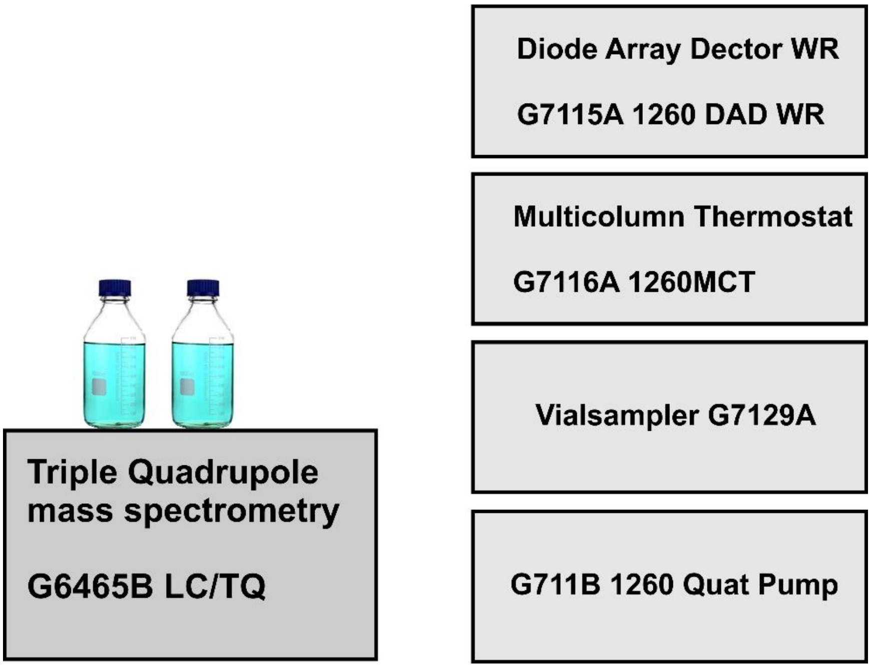

Figure S2. The schematic representation of the combination of HPLC and MS

PFAS analysis was conducted using HPLC (Agilent 1260 Infinity II)-MS/MS (G6465B) equipped with an Agilent Eclipse Plus C18 column (3.0 mm × 50 mm, 1.8 µm). Detection was performed in negative electrospray ionization (ESI) mode, employing dynamic multiple-reaction monitoring (dMRM) for quantitative analysis. The acquisition method was adapted from the demonstration protocol provided by Wellington Laboratories. Detailed information on the HPLC mobile phase gradient and the acquisition parameters for the triple quadrupole mass spectrometer (QQQ-MS) are presented in Tables S5 and S6. Calibration was performed using seven standard concentrations (0.5, 1, 5, 10, 30, 50, and 100 ng/mL), yielding a linear calibration curve. The method's detection range spanned from a lower limit of quantification (LOQ) of 0.5 ng/mL to an upper limit of 100 ng/mL.

Table S5. The mobile phase gradient of HPLC

**Mobile phase**	**A**	**B**
**Composition**	**10 mM Am-Ac in Water**	**10 mM Am-Ac in 80:20 MeOH:ACN**

Start. Cond. min	60.0 %	40.0%
1.00 min	60.0 %	40.0%
10.00 min	30.0 %	70.0%
14.00 min	10.0 %	90.0%
25.00 min	10.0 %	90.0%
26.00 min	60.0 %	40.0%
32.00 min	60.0 %	40.0%

Notes: ACN is Acetonitrile, and Am-Ac denotes Ammonium acetate.

Table S6. The determination parameters of QQQ MS

Group	Compound name	ISTD? Precursor	(m/z)	MS1 res	Product (m/z)	MS2 res	Dwell (ms)	Fragmentor (V)	CE (V)	Polarity	Ion Mode	RT (min)	RT window (min)
PFAAs	M4PFBA	Yes	217	Unit	172	Wide	100	60	6	-	ESI	3	8
PFAAs	PFBA	No	213	Unit	168.9	Wide	100	60	6	-	ESI	3	8
PFAAs	PFBA_SG	No	216	Unit	172	Wide	100	60	6	-	ESI	6	8
PFAAs	M3PFBS	Yes	302	Unit	80	Wide	100	120	30	-	ESI	13	8
PFAAs	PFBS	No	299	Unit	98.9	Wide	100	120	32	-	ESI	7	8
PFAAs	PFBS	No	299	Unit	80	Wide	100	120	40	-	ESI	7	8
PFAAs	M5PFHxA	Yes	318	Unit	273	Wide	100	68	16	-	ESI	15.2	8
PFAAs	PFHxA	No	311	Unit	268	Wide	100	72	4	-	ESI	15.2	8
PFAAs	PFHxA	No	311	Unit	119	Wide	100	72	20	-	ESI	15.2	8
PFAAs	PFHxA_SG	No	315	Unit	270	Wide	100	72	4	-	ESI	15.2	8
PFAAs	M3PFHxS	Yes	402	Unit	80	Wide	100	100	34	-	ESI	12.8	8
PFAAs	PFHxS	No	399	Unit	99	Wide	100	100	34	-	ESI	16	8
PFAAs	PFHxS	No	399	Unit	80	Wide	100	100	37	-	ESI	16	8
PFAAs	PFHxS_SG	No	403	Unit	103	Wide	100	100	34	-	ESI	12.8	8
PFAAs	M8PFOA	Yes	421	Unit	376	Wide	100	69	3	-	ESI	18	8
PFAAs	PFOA	No	413	Unit	369	Wide	100	69	3	-	ESI	18	8
PFAAs	PFOA	No	413	Unit	169	Wide	100	69	9	-	ESI	18	8

PFAAs	PFOA_SG	No	417	Unit	372	Wide	100	69	3	-	ESI	18	8
PFAAs	M8PFOS	Yes	507	Unit	80	Wide	100	100	38	-	ESI	15	8
PFAAs	PFOS	No	499	Unit	99	Wide	100	100	38	-	ESI	15	8
PFAAs	PFOS	No	499	Unit	80	Wide	100	100	38	-	ESI	15	8
PFAAs	PFOS_SG	No	503	Unit	99	Wide	100	100	38	-	ESI	17	8

Notes: The retention time (RT) and RT window for each compound are changeable depending on the separation column and pressure. CV is collision energy**.** SG is the abbreviation of the surrogate (the injection standard).

Table S7. The demonstration of instrumental analysis accuracy

n = 2	PFBA	PFBS	PFHxA	PFHxS	PFOA	PFOS
QC1 = 10 ng/mL	11.61	11.82	11.02	11.76	12.35	12.25
QC2= 30 ng/mL	33.25	33.59	31.68	31.55	33.89	29.59
Accuracy, %	116.13	118.22	110.15	117.59	123.51	122.53
Accuracy, %	110.82	111.98	105.59	105.16	112.96	98.63
Mean, %	113.48	115.10	107.87	111.37	118.23	110.58
S.D.	3.75	4.41	3.23	8.79	7.46	16.90

S5. Sequential Leaching Experiment Scheme

Ten grams (dry weight) of compost (PSD ⩽ 2.0 mm) were placed in a 500 mL centrifuge bottle, and 100 mL of elution solvent was added. The total mass was recorded, and the bottle was vortexed and then shaken for predetermined durations across four sequential stages: Stage I (10 minutes repeated for six steps), Stage II (1 hour repeated for five steps), Stage III (6 hours repeated for three steps), and Stage IV (12 hours repeated for two steps) (Figure S3). After each step, the mixture was centrifuged at 6000 RCF, and ~80 mL of the supernatant was collected into two 50 mL polypropylene Falcon tubes. The remaining compost was replenished with fresh solvent to restore the original total mass, followed by vortexing and shaking for the next step.

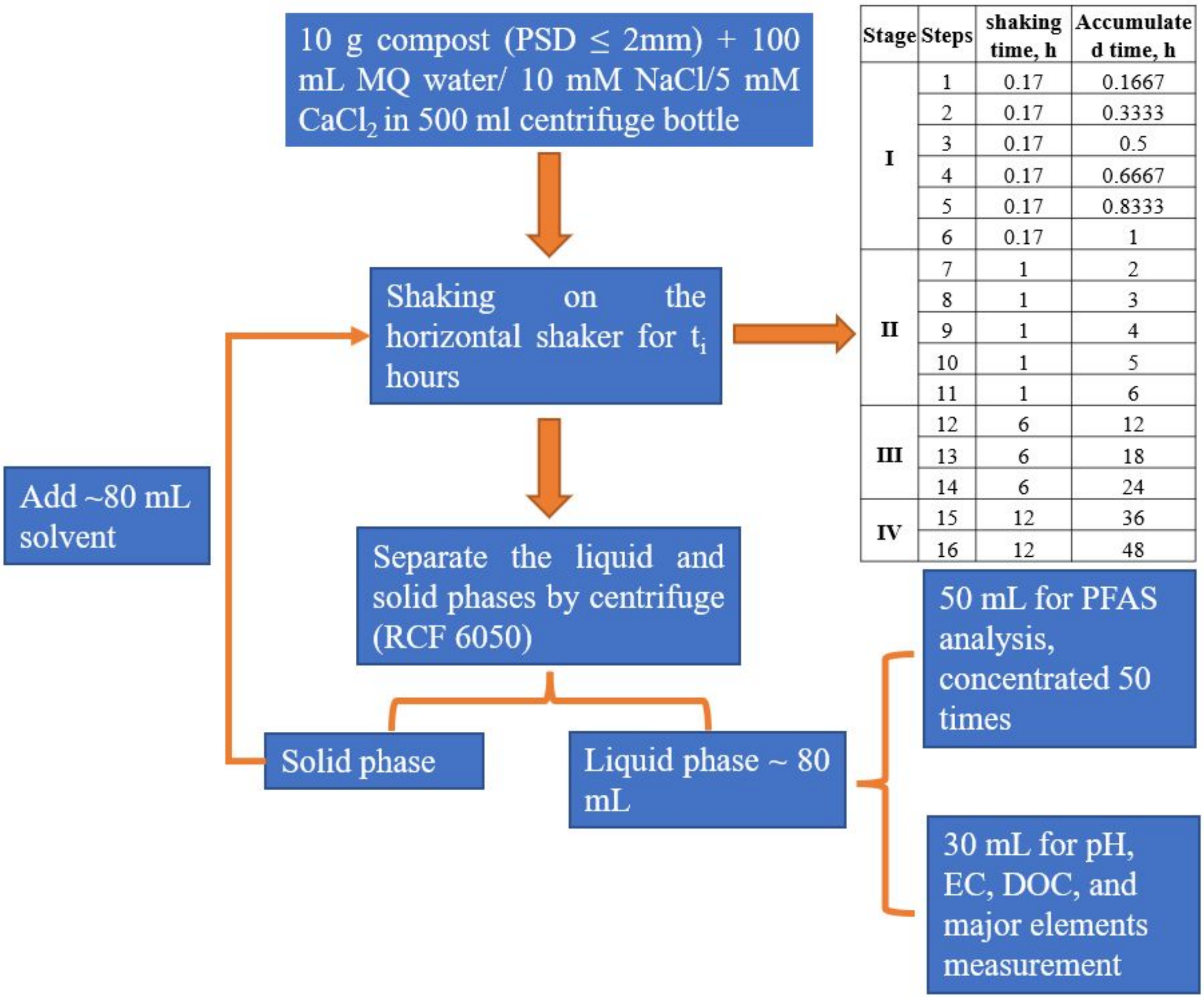

Stage	Steps	shaking time, h	Accumulated time, h
I	1	0.17	0.1667
	2	0.17	0.3333
	3	0.17	0.5
	4	0.17	0.6667
	5	0.17	0.8333
	6	0.17	1
II	7	1	2
	8	1	3
	9	1	4
	10	1	5
	11	1	6
III	12	6	12
	13	6	18
	14	6	24
IV	15	12	36
	16	12	48

Figure S3. The workflow of the PFAS release experiment. The release occurred in 16 steps; the accumulated release duration is 48 hours. PSD is particle size distribution. The release experiment consists of four stages: stage I: steps 1 - 6; stage II: steps 7 – 11; stage III: steps 12 - 14; stage IV: steps 15 - 16.

S6. Results

S6.1. Compost Characteristics

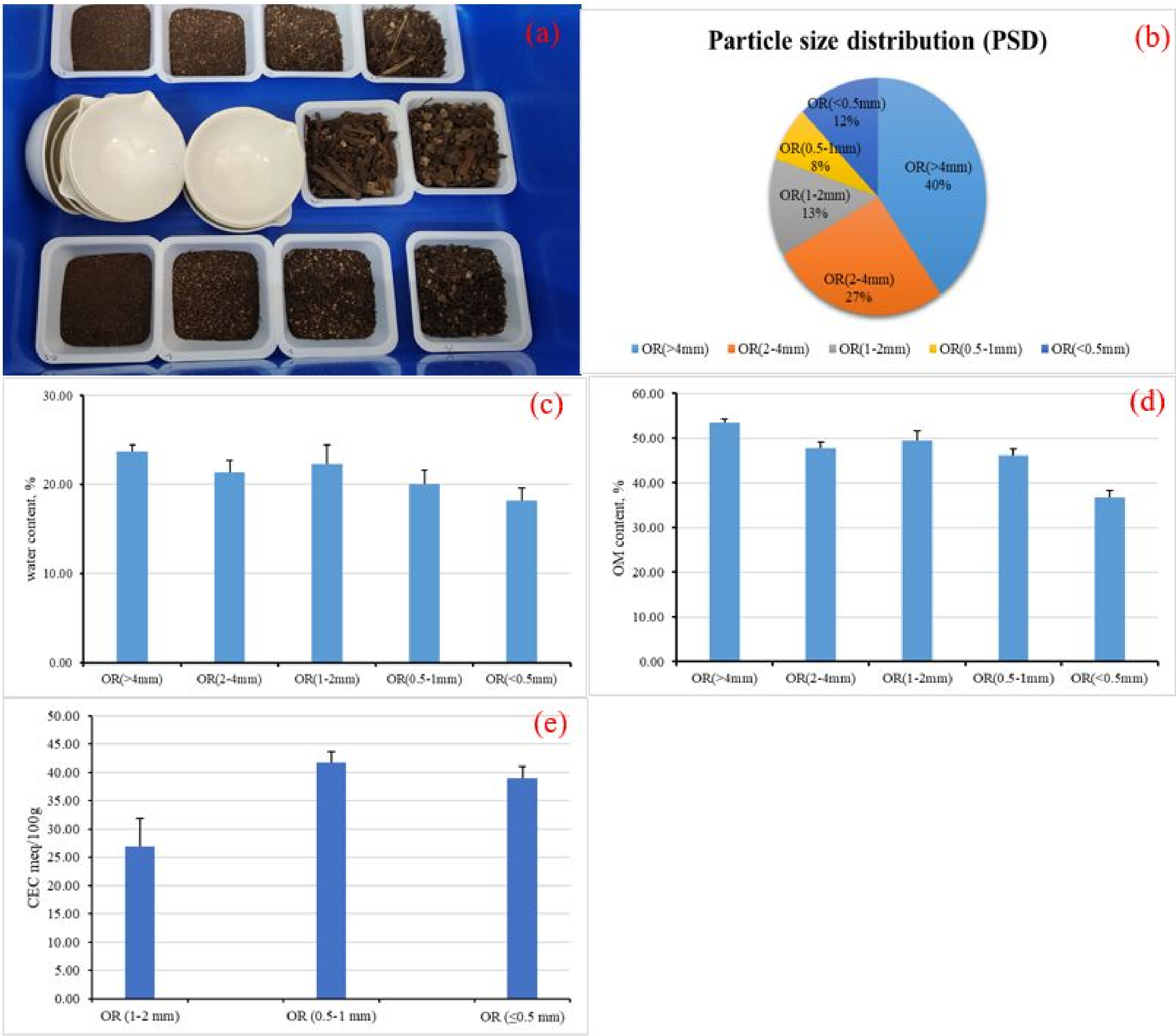

Figure S4. (a) The biosolid-derived compost (Or) of different PSD, from left to right <0.5 mm, 0.5 - 1 mm, 1 - 2mm, 2 - 4 mm, the two subplots in the middle are >4 mm; **(b)** The particle size distribution (PSD); **(c)** Water content; **(d)** The organic matter proportion; **(e)** the cation exchange capacity (CEC).

Table S8. Concentration of selected PFAS in the biosolid-derived compost

Compound	Molecular Formula	Concentration (ng/g)	Solubility in Water (25 °C)[a]
PFBA	$CF_3(CF_2)_2COOH$	139.9 ± 3.3	214 g/L
PFBS	$CF_3(CF_2)_3SO_3H$	195.6 ± 6.3	400–500 g/L
PFHxA	$CF_3(CF_2)_4COOH$	7.3 ± 0.2	15.7 g/L
PFHxS	$CF_3(CF_2)_5SO_3H$	5.9 ± 0.2	20–100 g/L
PFOA	$CF_3(CF_2)_6COOH$	11.9 ± 0.1	9.5 g/L
PFOS	$CF_3(CF_2)_7SO_3H$	63.3 ± 6.3	570 mg/L

Notice: [a] data was cited from https://pfas-1.itrcweb.org/

S6.2. Chemical Properties of the Eluted Solution

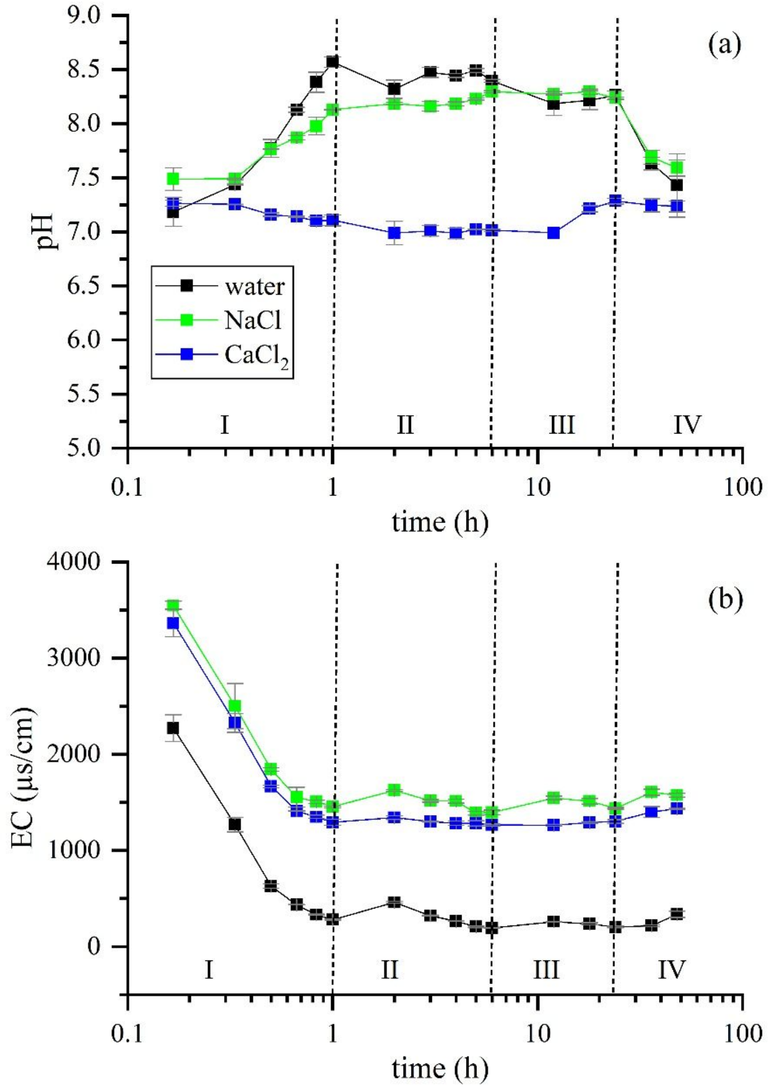

Figure S5. The **(a)** pH and **(b)** EC of the eluted solution from the release experiment with the elution solvent of water, 10 mM NaCl, and 5 mM $CaCl_2$.

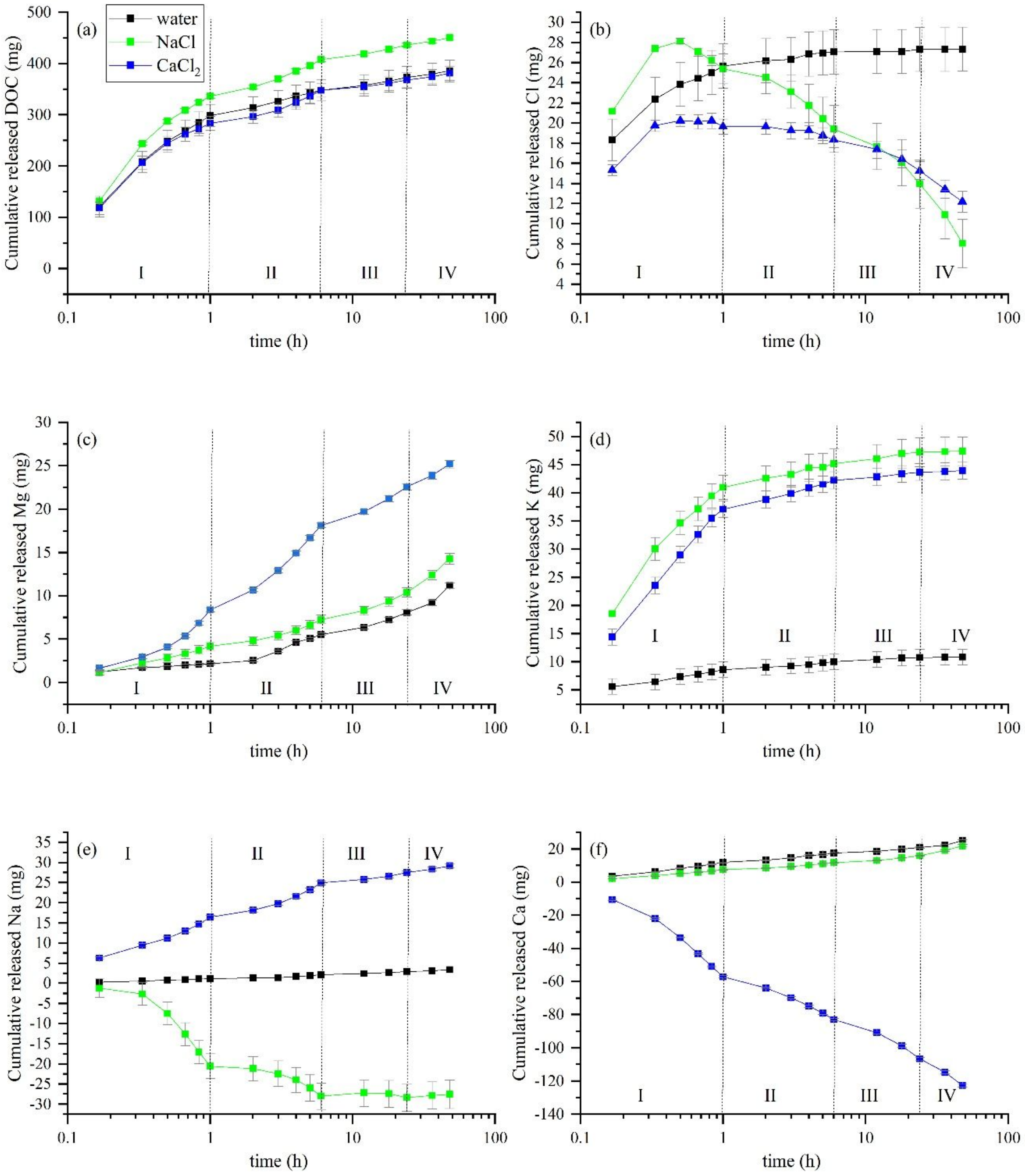

Figure S6. Cumulative released mass of **(a)** DOM (presented as DOC) and major ions, including: **(b)** Cl^-, **(c)** Mg^{2+}, **(d)** K^+, **(e)** Na^+, and **(f)** Ca^{2+}.

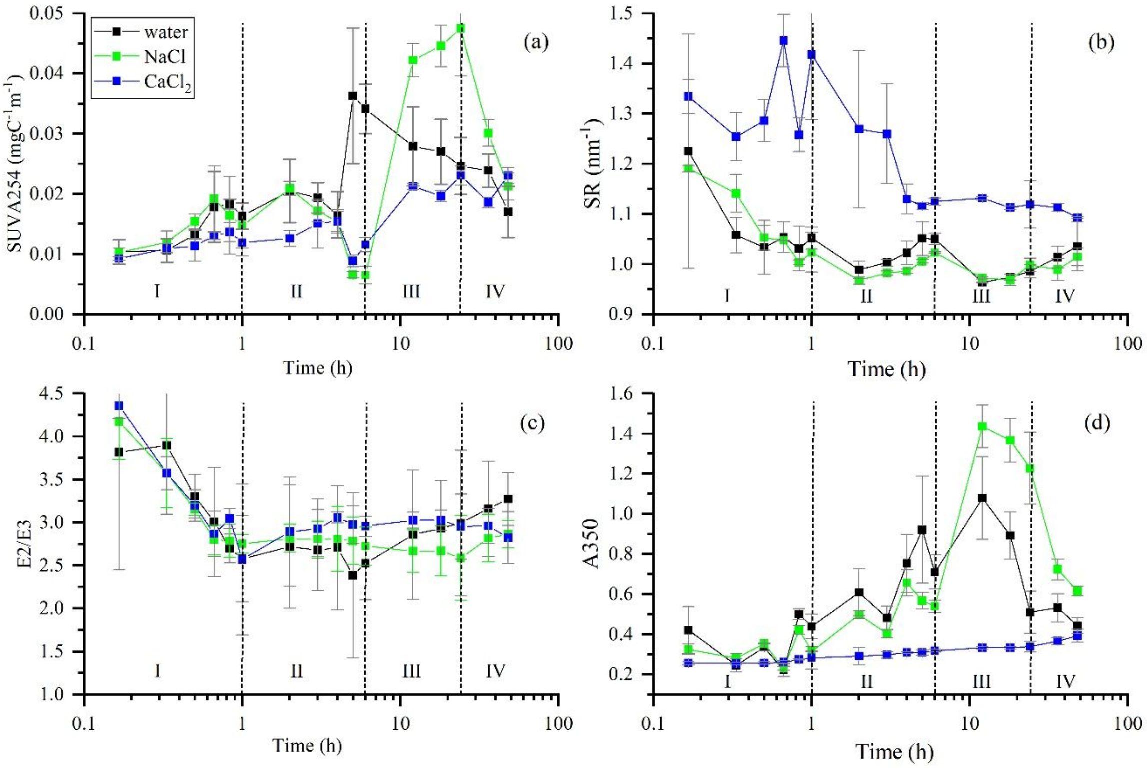

Figure S7. Characterizing DOM in the eluted solution by spectrophotometry, including: **(a)** SUVA254, **(b)** spectral slope ratio (SR), **(c)** E2/E3, and **(d)** A350.

SUVA254 = A254/DOC;

E2/E3 (250/365) = A250/A365.

A254, A250, A350, and A365 are the absorbances at 254 nm, 250 nm, and 365 nm.

SR = $S_{275\text{-}295}/S_{350\text{-}400}$, where $S_{275\text{-}295}$ and $S_{350\text{-}400}$ are the slopes of the absorbance spectrum between the wavelengths of 275-295 and 350-400. It was obtained by fitting the equation: $\log(230.3\times A_{\lambda}) = S_{\lambda}\times \lambda + b$ with the spectrum.

S6.3. Data of PFAS Release Kinetics

Table S9. The data of PFAS release kinetics from biosolid-derived compost with elution solvent of water

		**unit: ng/L**	**PFBA**		**PFBS**		**PFHxA**		**PFHxS**		**PFOA**		**PFOS**	
**Steps**	**Solution extracted (mL)**	**Solution remains in the bottle (mL)**	**Mean**	**S.D.**	**Mean**	**S.D.**	**Mean**	**S.D.**	**Mean**	**S.D.**	**Mean**	**S.D.**	**Mean**	**S.D.**
1	80.85	19.95	2606.18	102.63	1547.97	55.84	238.14	6.16	24.27	2.63	74.70	2.17	335.34	25.64
2	81.47	19.36	2237.75	120.32	1613.75	90.97	160.43	1.79	31.74	4.88	86.38	1.41	491.38	16.57
3	79.85	20.99	832.80	176.82	1254.04	51.92	57.18	1.33	79.62	19.86	63.95	2.18	568.78	35.89
4	82.61	18.57	292.05	0.00	1193.93	0.00	14.53	0.00	90.48	0.00	41.31	0.00	493.31	0.00
5	79.34	21.52	239.10	46.10	1063.59	51.73	13.65	9.10	76.71	10.97	44.07	13.61	576.88	0.30
6	80.56	20.27	162.18	25.93	884.23	73.53	0	0	55.47	5.07	33.54	2.81	442.91	3.09
7	85.56	16.51	180.74	21.65	650.03	191.95	0	0	52.52	17.01	33.81	6.71	368.47	34.87
8	84.26	16.82	145.75	23.51	706.39	83.83	0	0	41.77	2.95	28.14	8.34	322.46	52.70
9	84.39	16.42	108.97	14.95	547.06	1.07	0	0	36.58	0.09	25.00	4.87	323.42	13.93
10	85.01	15.84	89.68	21.12	566.45	58.47	0	0	26.22	6.61	24.35	7.36	287.52	64.85
11	81.44	19.15	92.62	12.37	359.34	12.26	0	0	24.84	3.29	23.01	3.84	235.21	14.33
12	84.59	15.97	192.61	24.52	335.19	38.13	0	0	18.90	3.78	24.91	8.55	210.89	44.26
13	85.15	15.63	167.25	36.58	298.38	39.65	0	0	17.60	5.41	25.06	6.34	201.28	42.24
14	83.52	17.01	149.61	28.00	312.07	8.07	0	0	14.45	1.71	23.85	6.06	181.91	6.60
15	85.11	15.41	152.75	40.19	250.94	22.40	0	0	8.72	1.42	23.28	7.12	155.22	26.10
16	86.14	14.65	142.29	30.52	150.98	13.58	0	0	0.00	0.00	22.67	7.03	111.73	5.45
Sample preparation blank, n = 2			0.19	0.09	0.02	0.01	0.02	0.02	0.02	0.02	0.15	0.06	0.13	0.06
Instrumental blank, n = 3			0.37	0.01	0.11	0.02	0.01	0.00	0.01	0.01	0.13	0.02	0.11	0.02

Notes: S.D. standard deviation.

Table S10. The data of PFAS release kinetics from biosolid-derived compost with elution solvent of 10 mM NaCl

		**unit: ng/L**	**PFBA**		**PFBS**		**PFHxA**		**PFHxS**		**PFOA**		**PFOS**	
**Steps**	**Solution extracted, mL**	**Solution reminds in bottle, mL**	**Mean**	**S.D.**	**Mean**	**S.D.**	**Mean**	**S.D.**	**Mean**	**S.D.**	**Mean**	**S.D.**	**Mean**	**S.D.**
1	77.02	21.22	2485.88	92.90	2185.14	261.93	186.46	0.68	22.21	1.28	49.78	2.66	157.24	15.89
2	76.59	21.62	1958.30	49.41	2110.29	40.12	151.69	6.91	21.19	0.88	53.06	0.10	190.18	10.11
3	78.70	20.56	826.00	58.57	1472.57	33.19	56.35	0.15	26.36	0.00	47.78	7.78	233.69	15.09
4	83.35	19.41	378.63	43.22	1083.87	19.24	21.66	1.30	18.38	0.53	37.86	3.93	228.33	11.00
5	78.01	21.80	242.55	26.66	1586.42	64.24	10.10	2.39	12.87	1.59	30.87	4.56	204.60	13.89
6	78.57	20.76	181.85	29.04	1590.82	344.57	0.00	0.00	6.87	4.51	25.49	3.64	193.95	15.36
7	79.61	19.32	269.81	28.15	1556.57	111.26	0.00	0.00	9.18	0.89	28.22	3.89	223.83	19.56
8	78.98	19.30	201.89	31.70	1203.51	114.68	0.00	0.00	0.00	0.00	24.85	2.39	221.79	20.99
9	81.66	17.17	181.66	24.75	906.14	71.89	0.00	0.00	0.00	0.00	20.76	2.93	190.16	4.68
10	83.18	16.22	89.89	23.67	629.50	2.30	0.00	0.00	0.00	0.00	17.50	2.93	183.59	34.25
11	83.22	14.97	85.68	0.10	843.83	66.08	0.00	0.00	0.00	0.00	17.04	0.27	167.26	1.34
12	82.60	15.61	97.25	11.07	823.20	86.17	0.00	0.00	0.00	0.00	17.97	5.24	136.26	29.91
13	82.82	15.71	123.60	0.27	816.65	40.29	0.00	0.00	0.00	0.00	16.65	3.34	106.08	12.87
14	82.41	15.78	87.92	8.05	719.66	6.24	0.00	0.00	0.00	0.00	14.34	3.40	92.02	6.52
15	84.41	15.37	73.68	22.20	495.87	55.72	0.00	0.00	0.00	0.00	13.52	1.40	64.72	6.96
16	79.90	18.61	120.66	12.52	453.96	1.45	0.00	0.00	0.00	0.00	13.97	1.41	49.45	6.10
Sample preparation blank, n = 2			0.47	0.01	0.44	0.00	0.05	0.00	0.01	0.01	0.34	0.00	0.35	0.02
Instrumental blank, n = 3			0.10	0.00	0.04	0.01	0.01	0.00	0.00	0.00	0.07	0.01	0.13	0.07

Notes: S.D. standard deviation.

Table S11. The data of PFAS release kinetics from biosolid-derived compost with elution solvent of 5 mM $CaCl_2$

		unit: ng/L	PFBA		PFBS		PFHxA		PFHxS		PFOA		PFOS	
Step	Solution extracted, mL	Solution reminds in bottle, mL	Mean	S.D.	Mean	S.D.	Mean	S.D.	Mean	S.D.	Mean	S.D.	Mean	S.D.
1	77.02	21.22	1128.97	202.31	867.07	27.23	200.81	26.46	14.03	3.52	45.96	1.97	162.98	8.82
2	76.59	21.62	1324.05	75.17	900.90	159.82	119.10	7.22	16.34	3.30	45.68	3.08	162.71	15.23
3	78.70	20.56	571.28	60.45	714.32	73.26	44.75	3.85	11.14	2.78	38.75	3.84	147.05	10.40
4	83.35	19.41	254.38	42.44	612.13	145.59	18.16	1.78	0.00	0.00	30.28	2.81	125.04	6.06
5	78.01	21.80	156.37	20.84	509.26	78.06	8.52	1.56	0.00	0.00	25.18	1.68	105.11	1.14
6	78.57	20.76	111.78	4.40	455.15	66.91	0.00	0.00	0.00	0.00	22.01	1.58	102.74	5.01
7	79.61	19.32	193.65	46.18	529.76	91.09	0.00	0.00	0.00	0.00	21.78	2.50	112.77	7.80
8	78.98	19.30	150.63	19.03	425.33	57.07	0.00	0.00	0.00	0.00	21.55	0.22	99.23	1.97
9	81.66	17.17	104.94	20.59	288.25	118.10	0.00	0.00	0.00	0.00	19.84	4.21	117.22	8.95
10	83.18	16.22	89.23	18.47	225.18	10.78	0.00	0.00	0.00	0.00	23.96	3.32	106.29	0.30
11	83.22	14.97	64.73	12.37	207.47	56.35	0.00	0.00	0.00	0.00	20.10	1.85	102.62	11.33
12	82.60	15.61	99.61	23.05	231.34	21.66	0.00	0.00	0.00	0.00	21.94	4.80	112.96	15.85
13	82.82	15.71	124.99	22.13	275.12	26.08	0.00	0.00	0.00	0.00	21.76	1.25	102.59	4.78
14	82.41	15.78	116.75	27.25	329.59	10.67	0.00	0.00	0.00	0.00	19.03	1.54	95.03	1.20
15	84.41	15.37	99.17	21.66	292.32	28.69	0.00	0.00	0.00	0.00	17.60	1.01	87.04	2.48
16	79.90	18.61	116.70	28.36	302.89	22.94	0.00	0.00	0.00	0.00	17.18	0.83	81.43	7.10
Sample preparation blank, n = 2			0.33	0.00	0.08	0.00	0.04	0.00	0.01	0.00	0.46	0.00	0.41	0.02
Instrumental blank, n = 3			0.05	0.00	0.03	0.00	0.01	0.00	0.00	0.00	0.10	0.03	0.16	0.01

Notes: S.D. standard deviation.

S6.4. The Optimized Model Parameters

Table S12. The optimized first-order two-compartment model parameters

		First-order two-compartment model (FOTWCM)								
**PFAS**	**Solvent**	F_1	**S.D.**	F_2	**S.D.**	k_1	**S.D.**	k_2	**S.D.**	R^2 (COD)
**PFBA**	**Water**	0.386	0.005	0.614	0.005	4.203	0.253	0.003	0.000	0.973
	**10 mM NaCl**	0.364	0.005	0.636	0.005	3.688	0.247	0.002	0.000	0.964
	**5 mM $CaCl_2$**	0.218	0.004	0.782	0.004	3.051	0.211	0.002	0.000	0.973
**PFBS**	**Water**	0.405	0.010	0.595	0.010	1.358	0.098	0.004	0.001	0.982
	**10 mM NaCl**	0.555	0.015	0.445	0.015	1.152	0.084	0.016	0.002	0.988
	**5 mM $CaCl_2$**	0.214	0.004	0.786	0.004	1.364	0.074	0.003	0.000	0.991
**PFHxA**	**Water**	0.542	0.002	0.458	0.002	6.017	0.166	0.000	0.000	0.987
	**10 mM NaCl**	0.455	0.003	0.545	0.003	5.466	0.236	0.000	0.000	0.975
	**5 mM $CaCl_2$**	0.416	0.000	0.584	0.000	6.432	0.080	0.000	0.000	0.997
**PFHxS**	**Water**	0.720	0.035	0.280	0.035	0.884	0.108	0.016	0.007	0.971
	**10 mM NaCl**	0.162	0.003	0.838	0.003	1.979	0.117	0.000	0.000	0.979
	**5 mM $CaCl_2$**	0.055	0.001	0.945	0.001	4.800	0.577	0.000	0.000	0.872
**PFOA**	**Water**	0.298	0.007	0.702	0.007	1.506	0.115	0.005	0.001	0.983
	**10 mM NaCl**	0.216	0.006	0.784	0.006	1.314	0.101	0.002	0.000	0.983
	**5 mM $CaCl_2$**	0.186	0.007	0.814	0.007	1.271	0.135	0.003	0.000	0.975
**PFOS**	**Water**	0.531	0.019	0.469	0.019	0.971	0.094	0.011	0.002	0.981
	**10 mM NaCl**	0.264	0.011	0.736	0.011	0.688	0.071	0.003	0.001	0.982
	**5 mM $CaCl_2$**	0.150	0.006	0.850	0.006	0.980	0.116	0.003	0.000	0.976

Notes: S.D. standard deviation; R^2: coefficient of determination.

S6.5. One-Way ANOVA test of PFAS release kinetics

The one-way ANOVA was conducted to assess differences in PFAS release kinetics between eluents during two distinct phases: Fast (first hour) and Slow (remaining duration). The PFAS concentration data in compost (ng/kg) used for the test were derived from Section S6.3 and the results are summarized in Table S13. For each PFAS, four comparisons were performed: t123 (water vs. NaCl vs. $CaCl_2$), t_{12} (water vs. NaCl), t_{13} (water vs. $CaCl_2$), and t_{23} (NaCl vs. $CaCl_2$).

The resulting p-values for each PFAS are summarized in the table below. A p-value $\leqslant$ 0.05 indicates a statistically significant difference and is considered meaningful. In the Fast release phase, at least two groups are significantly different. However, most of the statistically significant differences occurred in the slow phase. Most p-values were zero or close to zero, indicating no further release or variability in this stage. These prove that the effects of 10 mM NaCl and 5 mM $CaCl_2$ are significantly different.

Table S13. The results of the one-way ANOVA test for PFAS release kinetics

PFAS	Eluent	Fast	Slow
PFBA	t123	**0.0005**	**3.48E-20**
	t12	0.0906	**1.45E-09**
	t13	**0.0008**	**2.24E-15**
	t23	**0.0022**	**5.39E-13**
PFBS	t123	**0.0278**	**1.22E-12**
	t12	0.7916	**0.0130**
	t13	**0.0231**	**9.87E-11**
	t23	**0.0105**	**3.55E-10**
PFHxA	t123	**0.0040**	0.0000
	t12	**0.0185**	0.0000
	t13	**0.0061**	0.0000
	t23	0.5204	0.0000
PFHxS	t123	**0.0094**	**4.03E-23**
	t12	0.0503	**1.01E-14**
	t13	**0.0179**	**1.01E-15**
	t23	**0.0162**	0.0000
PFOA	t123	**0.0254**	**8.36E-10**
	t12	0.0604	**4.09E-07**
	t13	**0.0256**	**1.68E-07**
	t23	0.5396	0.2254

PFOS	t123	**0.0064**	**3.07E-15**
	t12	**0.0299**	**1.38E-09**
	t13	**0.0136**	**2.48E-11**
	t23	0.3340	**0.0001**

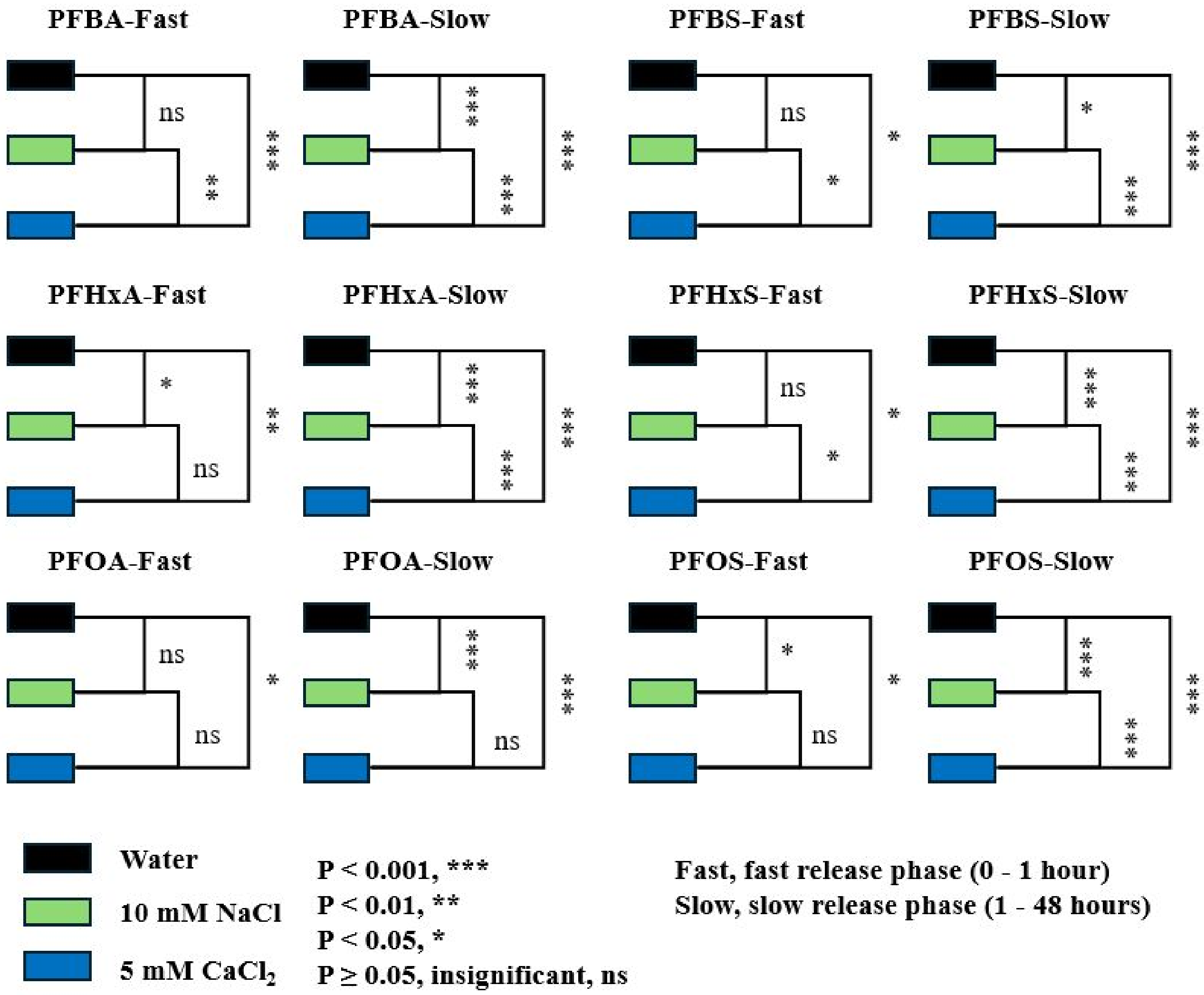

Figure S8. The schematic presentation of the results of the one-way ANOVA test

S6.6. Correlation Analysis Between PFAS, DOM, and Inorganic Ions

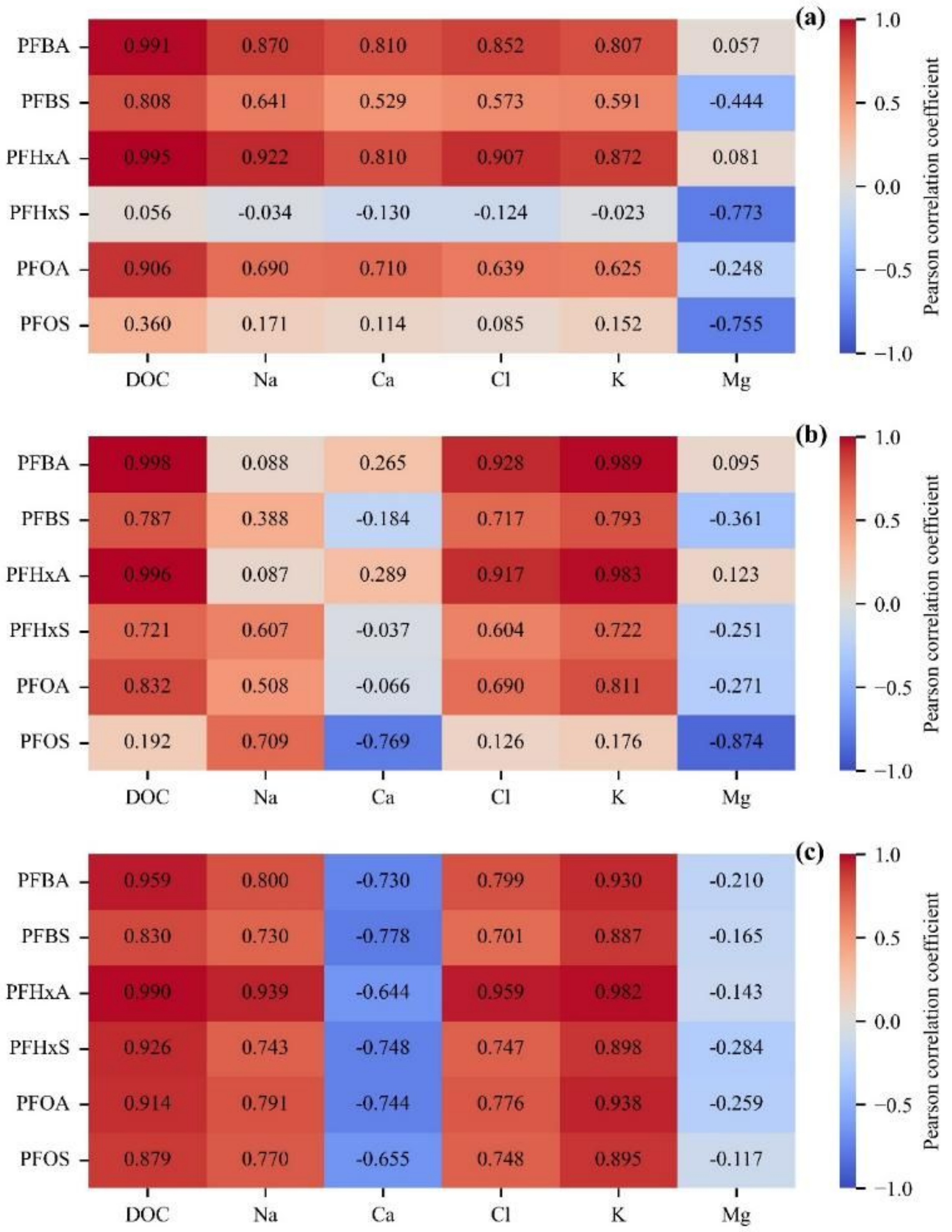

Figure S9. Pearson correlation coefficient of the release kinetics between PFAS, DOC, and ions (including Na, Ca, Cl, K, and Mg). (a) water, (b) 10 mM NaCl, and (c) 5 mM $CaCl_2$.
